# A New Biomaterial Derived from *Aloe vera*—Acemannan from Basic Studies to Clinical Application

**DOI:** 10.3390/pharmaceutics15071913

**Published:** 2023-07-09

**Authors:** Yingjie Bai, Yimeng Niu, Shengao Qin, Guowu Ma

**Affiliations:** 1 School of Stomatology, Dalian Medical University, No. 9 West Section, Lvshunnan Road, Dalian 116044, China; 2 Academician Laboratory of Immune and Oral Development & Regeneration, Dalian Medical University, Lvshun South Road, Dalian 116044, China; 3 Department of Stomatology, Stomatological Hospital Affiliated School, Stomatology of Dalian Medical University, NO. 397 Huangpu Road, Shahekou District, Dalian 116086, China

**Keywords:** biomaterials, acemannan, polysaccharide, *Aloe vera*, tissue regeneration, dental regeneration, bone regeneration, wound healing

## Abstract

*Aloe vera* is a kind of herb rich in polysaccharides. Acemannan (AC) is considered to be a natural polysaccharide with good biodegradability and biocompatibility extracted from *Aloe vera* and has a wide range of applications in the biomedical field due to excellent immunomodulatory, antiviral, antitumor, and tissue regeneration effects. In recent years, clinical case reports on the application of AC as a novel biomedical material in tissue regenerative medicine have emerged; it is mainly used in bone tissue engineering, pulp–dentin complex regeneration engineering, and soft tissue repair, among other operations. In addition, multiple studies have proved that the new composite products formed by the combination of AC and other compounds have excellent biological and physical properties and have broader research prospects. This paper introduces the preparation process, surface structure, and application forms of AC; summarizes the influence of acetyl functional group content in AC on its functions; and provides a detailed review of the functional properties, laboratory studies, clinical cutting-edge applications, and combined applications of AC. Finally, the current application status of AC from basic research to clinical treatment is analyzed and its prospects are discussed.

## 1. Introduction

In the medical field, biomaterials are essential. They are materials that are implanted into the living system of an organism and combine with the living system but do not react with the organism. Biomaterials used in the medical field, also called biomedical materials, are mainly used to diagnose and treat body tissues and organs or to enhance their functions. They have good hemocompatibility, histocompatibility, and immunity and do not have adverse effects on human tissues [1]. There are various types of biomedical materials, which can be divided into natural biomaterials, synthetic polymer biomaterials, medical metal materials, inorganic biomedical materials and composite biomaterials, etc., according to their properties [2,3]. Natural biological materials are obtained from animals or plants by direct extraction, such as polysaccharides. Polysaccharides are organic components of all living organisms. As biomolecules, polysaccharides can be enzymatically hydrolyzed in living organisms into small molecule substances that can be easily absorbed by living organisms and have no toxic side effects. They are the most abundant biomedical materials in plants, and various polysaccharides have been widely used in the medical field [4]. For example, chitosan (CS), as a class of biodegradable materials approved by the FDA, is widely used in biomedical material fields such as direct pulp capping surgery to promote the formation of dentin bridge, tissue regeneration engineering to repair soft and hard tissue defects, and drug delivery systems to control the rate of drug release [5,6,7,8]. Through recent research, the continuous development of polysaccharides from plants into biomedical materials has revolutionized technology and concepts in the medical field, supporting the advancement and development of medical science.

**Figure 1:**
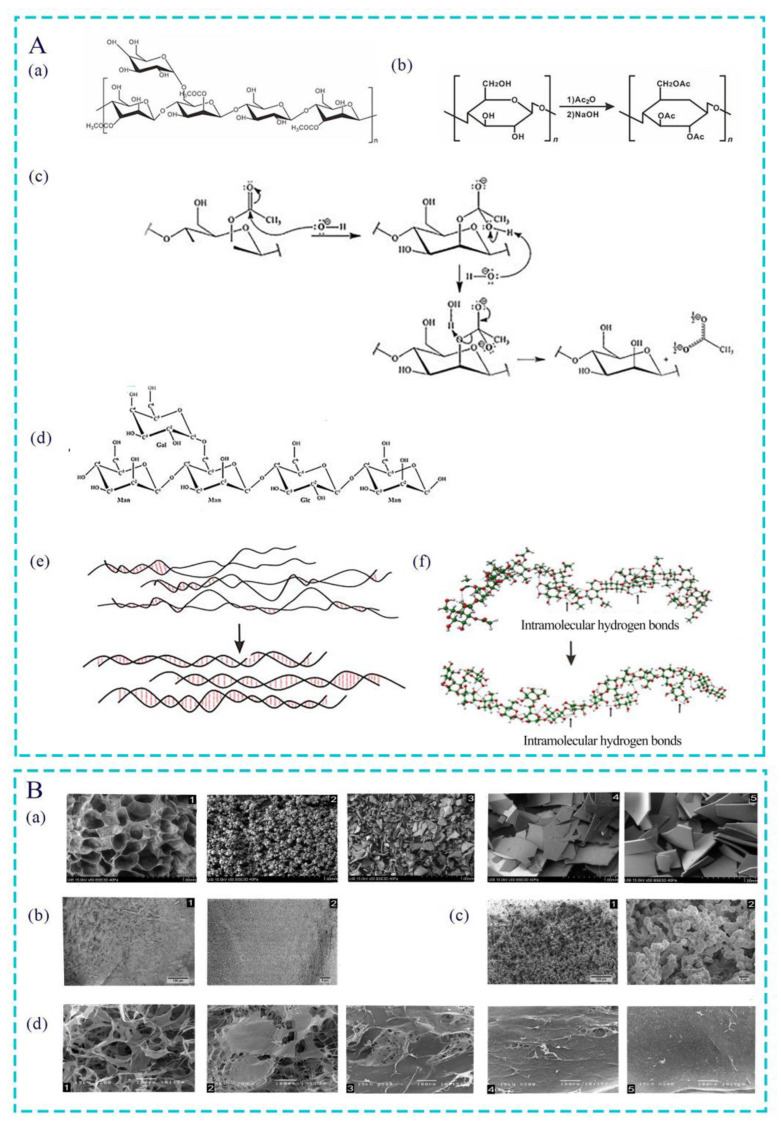
(**A**). Structure of acemannan (AC) and deacetylated acemannan (De-AC). Molecular structure of AC. (**a**) Acetylation process of polysaccharides (**b**). Deacetylation process of AC (**c**). Molecular structure of 100% De-AC (**d**). Deacetylation process of AC (based on a computational simulation) (**e**). Deacetylation process of AM1-optimized structures of AC double-stranded tetramer (**f**) [13]. (**B**). AC surface structure under different treatments. From left to right: *Aloe vera* reference, processed samples treated with spray-drying, industrial freeze-drying, refractance window-drying, and radiant zone-drying in order (**a**) [14]. Surface structure of M1 AC aerogel at 200 and 2000 scale bar (**b**). Surface structure of M2 AC aerogel at 200 and 2000 scale bar (**c**) [15]. Surface structure of AC under different degrees of deacetylation. From left to right: AC and 10%, 35%, 50%, and 100% De-AC in order (**d**) [13].

*Aloe vera*, as a succulent herbaceous plant in the Liliaceae family, contains over 300 varieties. Among them, *Aloe barbadensis* Mill. has higher economic and medicinal value and is most widely used in the medical field [9]. Polysaccharides are the main bioactive components of *Aloe barbadensis* Mill. The Carrington Laboratory in the United States named the acetylated mannan extracted and purified from *Aloe barbadensis* Mill. “acemannan (AC)”. AC is a highly acetylated mannan that is produced by a specialized cell called white matter and linked by β-(1,4) glycosidic bonds, which has undergone a series of extraction and purification processes for experimental and clinical research (Figure 1A(a)) [10,11]. It is widely believed that AC has the potential to treat various diseases, such as oral diseases, systemic metabolic diseases, cardiovascular system diseases, and benign and malignant tumors [12]. In recent years, clinical treatment cases using AC as biomaterials have emerged, especially in tissue regeneration. In addition, considerable progress has been made in the preparation of AC in combination with other compounds to form composite hydrogels, aerogels, membranes, and scaffolds. Therefore, this review summarizes the sources and preparation methods of AC, as well as the influence of acetyl groups on the function and activity of AC. Moreover, it provides a detailed introduction to the various application forms, functional characteristics, clinical cutting-edge therapeutic research, and combined applications of AC. Finally, this article analyzes the limitations of AC research and provides constructive suggestions, providing a scientific basis for the clinical use of AC in the treatment of multi-organ and systemic diseases.

## 2. Manufacturing Process of AC Products

### 2.1. Crude Extraction

The most common extraction method of AC is ethanol precipitation. The general process includes cleaning the leaves, homogenizing and centrifugating the *Aloe vera* gel, mixing with ethanol, and collecting precipitate to obtain AC particles [12,16]. It should be noted that, after centrifugation, the *Aloe vera* gel often contains a large amount of water and needs a further drying and dehydration procedure that does not affect the quality of the product. At present, a commonly used drying method is freeze-drying (FD), including industrial freeze-drying (IFD) and laboratory freeze-drying, which uses a low temperature to dry products to achieve dehydration [17,18]. Spray-drying (SD) [19,20], refractance window drying (RWD), and radiant zone drying (RZD) are also used [14,19]. However, the conditions applied in different drying processes can affect the yield, structure, physical and chemical properties, and even physiological and pharmacological properties of AC. For example, SD, IFD, and RWD were found to degrade galactose at the highest rate; SD, RWD, and RZD procedures reduced acetylation to varying degrees, with reduction rates of 70%, 52%, and 60%, respectively [14]. Moreover, under an electron microscope, it was observed that aloe gel was usually spongy with porous gaps, but its surface structure changed in different ways after different drying processes. For example, the surface structure of *Aloe vera* gel treated with SD and IFD was granular. The former was regularly spherical and/or oval with a smooth particle surface, while the latter was irregularly granular with uneven particle size. However, smooth flake morphology of uniform thickness was observed in *Aloe vera* gel treated with RWD or RZD (Figure 1B(a)).

### 2.2. Separation and Purification

To separate non-sugar substances from the mixed polysaccharide system, AC should be further isolated and purified. Ion-exchange column chromatography and gel permeation chromatography are the most commonly used methods for purifying AC. However, these methods are time-consuming, low in yield, and expensive, making them unsuitable for large-scale industrial applications. In recent years, more efficient and environmentally friendly separation and purification methods, such as the graded precipitation of organic reagents and ammonium sulfate, which can effectively separate a variety of plant polysaccharides into high-purity components according to the differences in molecular weight and structure between polysaccharides, have gradually become trendy [21,22,23]. Compared with column chromatography and precipitation methods, the membrane ultrafiltration method has low energy consumption, is simple to operate, and does not require the addition of chemicals. However, due to the high viscosity of aloe gel, membrane pollution is easily produced, which affects the quality of products and limits its application [24]. Moreover, the aqueous two-phase system with high selectivity can also be combined with membrane ultrafiltration for large-scale separation and purification of polysaccharides, which can greatly improve the membrane flux [24,25].

### 2.3. Structure Identification

**Figure 2:**
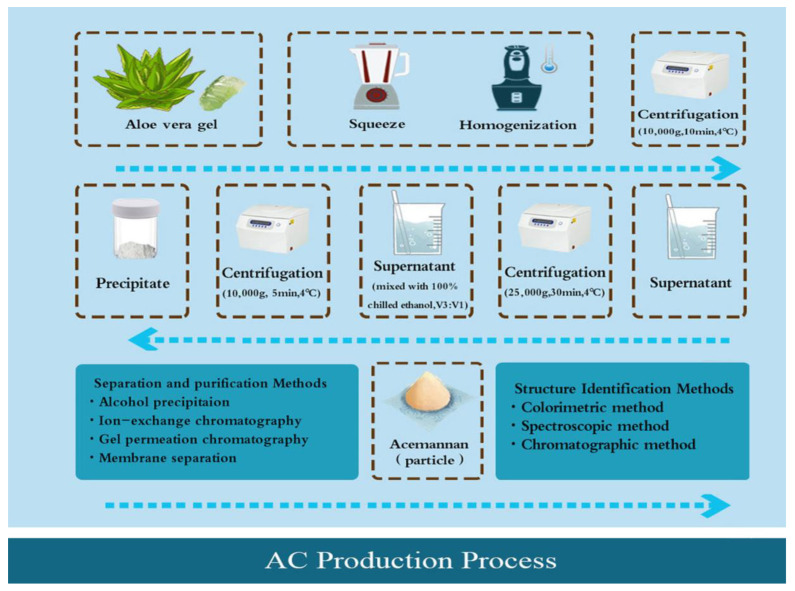
Process of extraction, separation purification, and structure identification of AC particle from *Aloe vera*.

To further clarify the homogeneity, molecular weight, acetyl group, and monosaccharide composition of the extracted AC, it is often necessary to identify its structure. Currently, the most commonly used methods for this are colorimetry, spectroscopy, and chromatography [16,26]. Colorimetric methods mainly include phenol–sulfuric assays and Congo red assays, which are commonly used to determine the content of polysaccharides in *Aloe vera* [27,28]. Spectral methods are often used to identify the structure of AC, such as Fourier-transform infrared spectroscopy [29], nuclear magnetic resonance spectroscopy [27], and ultraviolet–visible spectroscopy [30]. They can not only detect the configuration of acetyl functional groups and glycosidic bonds but can also infer the types of connecting bonds of the main chain and branch chain. Many chromatography methods, such as high-performance gel penetration chromatography [31], high-performance liquid chromatography [13], and gas–liquid chromatography [32], can also be used to determine the homogeneity and molecular weight of polysaccharides because they have high accuracy and sensitivity, attributes which are essential in clearly identifying the main sugar monomers released after the hydrolysis of polysaccharide acids. In recent years, some scholars have proposed a new method for extracting polysaccharides from *Aloe vera* using carbohydrate microarray profiling. According to the high-throughput ability of microarray technology and the specificity of molecular probes, the composition of cell wall polysaccharides from *Aloe vera* can be identified [33]. The complete extraction process of AC is shown in Figure 2.

## 3. Application Forms of AC

### 3.1. AC Particle

After lyophilizing and grinding, white AC particles can be obtained. Granular agents have many advantages, including the following: (I) Granular agents can dissolve in the blood, allowing drugs to quickly exert their effects. (II) When granular agents are applied to bone tissue regeneration, they can be attached to irregular bone surfaces and have a filling effect on large bone defects. (III) Granular agents have more stable properties than liquid and gas agents, can be stored in a dry environment at room temperature after ionizing radiation disinfection, and are convenient for transportation and carrying. (IV) The AC particle has low drug dispersion, adhesion, aggregation, and moisture absorption in the production process. The AC particle is the most direct form of drug for laboratory pharmaceuticals. Based on scanning electron microscopy (SEM), the average size of the particles was 32.45 mm, but particles that were too large or too small were frequently observed. Problems such as poor mixing, delamination, and secondary processing caused by different particle sizes still need to be solved [34].

### 3.2. AC Sponge

AC sponge is one of the most widely used forms in laboratory research and clinical treatment; examples include maxillary sinus lifting [34], bone defect filling [35], and pulp capping [36]. The specific production steps are as follows: dissolve granular AC in sterile double distilled water, freeze at −80 °C in a refrigerator, and freeze-dry for 24 h in a freeze-drying machine [37]. The main advantages of spongy agents are as follows: (I) SEM analysis shows that AC sponge has a pore structure with a diameter of 100~260 um, which provides a larger environment for the adhesion and growth of cells and tissues. When repairing periodontal tissue defects, the use of AC sponge is beneficial for the attachment and growth of periodontal ligament cells (PDLCs) [38]. (II) The spongy agent has a soft overall texture and has a filling effect on the pitted defect tissue. (III) After absorbing blood, the porous structure of the sponge agent produces volume expansion, which has an even mechanical compression effect on the bleeding wound. When the sponge touches the blood, it can absorb and agglutinate platelets and red blood cells, speeding up clotting. (IV) Sponges also act as a buffer against external tissue pressure. However, due to its porous structure, poor support strength and mechanical properties are also significant defects. This form should not be used for tissue defects in load-bearing areas such as articular cartilage and alveolar crest defects.

### 3.3. AC Hydrogel and Aerogel

Since mannan is a highly branched polymer with good water solubility, it can build a polysaccharide-based network skeleton in the gel material, thereby enhancing the mechanical strength and water absorption performance of the gel. Therefore, AC is suitable for making gel. In the preparation of AC hydrogel, the AC particles first need to be dissolved in distilled water to form a solution, and then the AC hydrogel can be obtained by using the physical or chemical cross-linking method. The physical cross-linking method refers to the hydrogel formed by intermolecular interaction, hydrogen bonding, crystallization, etc. For example, AC solution was placed in an alkali or non-solvent vapor environment. After vapor diffusion for 48 h, AC molecule self-assembly can be induced to form AC physical gel [15]. However, hydrogel prepared via the physical cross-linking method usually has poor mechanical properties, poor long-term stability, and is easily degradable. Chemical cross-linking refers to the cross-linking of polymer chain segments through covalent bonds, which generally requires the addition of cross-linking agents such as calcium chloride and polyvinyl alcohol (PVA) [39,40]. Compared with the physical cross-linking method, hydrogels synthesized by using the chemical cross-linking method have better mechanical strength and stability, but it can also easily cause initiator, cross-linking agent, and unreacted monomer residues. AC hydrogel can be developed as a wound dressing for the treatment of surgical wounds because of its good permeability, high porosity, high water content, and good biocompatibility [15] In addition, AC aerogel can be prepared from AC hydrogel via supercritical CO_2_ drying technology. Compared with AC hydrogel, AC aerogel has higher surface porosity, which is suitable for various adsorption/absorption of drugs, cell components, pollutants, liquids, and other substances. Therefore, AC aerogel has the potential for application in many fields, such as biomedicine, pharmaceutical, tissue engineering, and food [15]. However, it was found that the physical properties of the AC aerogels made by different preparation processes were different. For example, aerogel prepared by placing AC solution in an environment containing ammonium hydroxide (M1) exhibited a type IV isotherm with an H2-type hysteresis loop, which suggested a well-built mesoporous structure with a calculated specific surface area of about 370 m^2^/g. In contrast, aerogels prepared in an acetone environment (M2) exhibited higher density morphology, with a calculated specific surface area of about 10 m^2^/g. The surface structures of the two aerogels were also different. For example, the M1 aerogel showed a dense and obviously smooth surface with irregular depression under an electron microscope at low magnification, and an obvious mesoporous structure was observed after magnification; however, the M2 aerogel showed an obvious open porous structure at the lowest magnification (Figure 1B(b,c)).

### 3.4. AC Film

**Figure 3:**
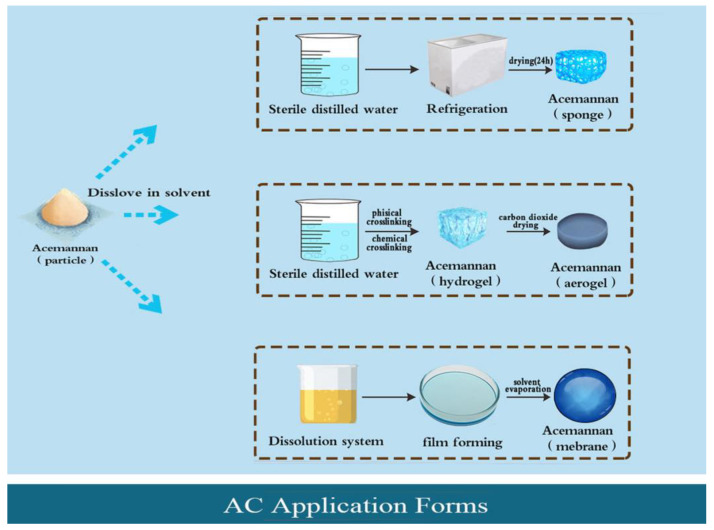
Production process of AC sponge, gel, and film.

In recent years, AC biofilms have gradually become the main material of bioactive scaffolds in tissue regeneration engineering. The preparation process usually uses solution casting, which involves dissolving AC particles in a polar solvent to produce a uniform film-making solution. Then, the solution is poured onto the glass plate to form a uniform thin layer with a certain thickness and is moved to a specific environment to make the solvent volatilize to form a uniform film. In addition, according to the preparation process, other forms of films can be made. For example, the film can be further cross-linked with calcium chloride as a cross-linking agent to obtain hydrogel films that can absorb and retain large amounts of water without dissolving. The composite film made by electrospinning AC and other compounds not only enhances the mechanical properties of the films but also is more conducive to exhibiting the biological activity of AC [41,42]. The production process of AC (sponge, gel, and film) is shown in Figure 3.

## 4. Effect of Acetyl Group on Bioactivity of AC

### 4.1. Acetylation Modification of Polysaccharide

Polysaccharides are one of the four basic substances that constitute life. They are natural high-molecular-weight polymers containing aldehyde or ketone groups, which are composed of more than ten monosaccharide molecules connected by the glycosidic bond in a linear or branched way [43]. Research has found that the biological activity of polysaccharide is closely related to its structure, i.e., its molecular weight, degree of branching, degree of polymerization, type of glycosidic bond and monosaccharide, and spatial conformation. All of these can affect the function of polysaccharides [44]. When the structure of polysaccharide molecules is modified by using physical or chemical methods, the physicochemical properties and biological activity of polysaccharides can be optimized. Among them, acetylation modification is a common chemical modification method used to modulate their hydrophobicity, processing properties, and biological activity and reduce the toxic effects of natural polysaccharides [45,46,47]. The acetylation modification method of polysaccharides is the acetic anhydride–pyridine method. The general steps include dissolving the polysaccharide sample in organic solvents such as pyridine, methanol, dimethyl sulfoxide, or formamide and then slowly adding acylation reagents such as acetic acid or acetic anhydride dropwise for acylation reaction. Finally, after the mixture is cooled and the pH adjusted, the acetylated polysaccharide compounds are obtained via precipitation with alcohol and dialysis [44]. The main mechanism is that, under appropriate conditions, the active hydroxyl groups of natural polysaccharides undergo nucleophilic substitution and generate corresponding polysaccharide esters. The reaction mechanism is shown in Figure 1A(b).

### 4.2. Effect of Acetyl Groups on Biological Activity of Polysaccharides

Numerous studies have found that some polysaccharides extracted directly from plants carry a large amount of acetyl groups, while others do not contain or contain very small amounts of acetyl groups. However, molecular modification methods, such as the acetylation modification method, can increase the acetyl group content. Acetylation modification is the insertion of acetyl groups into polysaccharide molecules, which changes their orientation and transverse order, causes changes in the spatial conformation of polysaccharide chains, exposes more polar groups, and alters their biological activity [44].

Acetyl groups are generally connected to polysaccharides through oxygen or nitrogen atoms, which largely affect the biological activity of polysaccharides. Firstly, as a good proton provider in polysaccharides, acetyl groups can inhibit free radical reactions and exert antioxidant activity. Compared to original polysaccharides, acetylated polysaccharides significantly enhance DPPH radicals, superoxide radicals, and total antioxidant capacity and have better protective effects on hydrogen-peroxide-induced cell oxidative damage [48,49]. Moreover, when the degree of acetyl substitution increases, even though the characteristic viscosity of the polysaccharide solution decreases, the antioxidant activity still increases [50]. Secondly, the acetylation-based treatment of polysaccharides greatly enhances their immune activity. For example, acetylated modified glucomannan not only promotes the adhesion of macrophages but also activates macrophages, inducing macrophages to secrete bone differentiation factors [51]. In addition, when the degree of acetylation increases to 1.8, the Toll-like receptor 2 (TLR2) signaling pathway can be specifically activated, thereby inducing macrophages to produce an antitumor phenotype and exert immune activity [52]. When the tremella acid polysaccharide and Ganoderma atrum polysaccharide were acetylated, the acetylated polysaccharide derivatives showed better effects on the activity of mouse spleen lymphocytes and mouse peritoneal macrophage plasma cells. It has been proven that polysaccharides exhibit immunomodulatory activity after acetylation modification, and the immunomodulatory activity is not only dependent on the presence of acetyl groups but also related to the degree of acetyl substitution [48,53]. In addition, acetyl groups also affect the antihyperlipidemic and anticoagulation activities of polysaccharides. After acetylation treatment, ulvan polysaccharides had stronger anti-hyperlipidemic activity, especially in reducing the level of triglycerides and low-density lipoprotein cholesterol levels [54]. Collectively, the polysaccharides modified via acetylation had significantly stronger anticoagulant ability because the introduction of acetyl groups can expose the polysaccharide branched chain hydroxyl group, enhance the water solubility of the polysaccharide, and thus enhance its anticoagulant activity [55,56].

### 4.3. Effect of Different Degrees of Acetylation Modification on AC

Acetyl is the basis for affecting the functional properties of polysaccharides and broadening their application range. Different degrees of acetylation will also have a certain impact on the structure and activity of AC in aloe. On the one hand, the deacetylation of AC will affect its molecular and surface structure. The deacetylation process of AC and the structure of completely deacetylated acemannan (De-AC) are shown in Figure 1A(c,d). X-ray diffractometry, SEM, and computational simulations showed that, after AC deacetylation, the intermolecular hydrogen bonds changed from a simple and loose irregular structure to a more regular and orderly structure (Figure 1A(e)). Complete deacetylation even eradicated the interrupted structure found in AC double-stranded tetramer (Figure 1A(f)). The surface structure of AC was also affected by acetyl groups. After deacetylation, the surface structure of AC changed from a sponge-like structure with multiple three-dimensional connecting pores to a flat layered structure with irregular pores. As the degree of deacetylation increased, AC showed a denser structure, and the porosity of the three-dimensional connection gradually disappeared (Figure 1B(d)). On the other hand, the biological activity of AC also changed due to the degree of acetylation. As the deacetylation effect increased, hydrophilicity, which induces the cell ability [13] and antibacterial and anti-biofilm potential of AC, decreased [57]. By contrast, as the degree of acetylation increased, the viscosity and thermal stability of AC increased. Moreover, the over-acetylated AC can reduce the oxidative damage and hematopoietic damage induced by γ radiation and enhance their immune regulatory effects by clearing free radicals and activating the secretion of hematopoietic factors, the latter of which is carried out by macrophages [58]. In summary, the degree of acetylation modification has a significant impact on AC.

## 5. Biological Functions of AC

### 5.1. Immunoregulation

**Figure 4:**
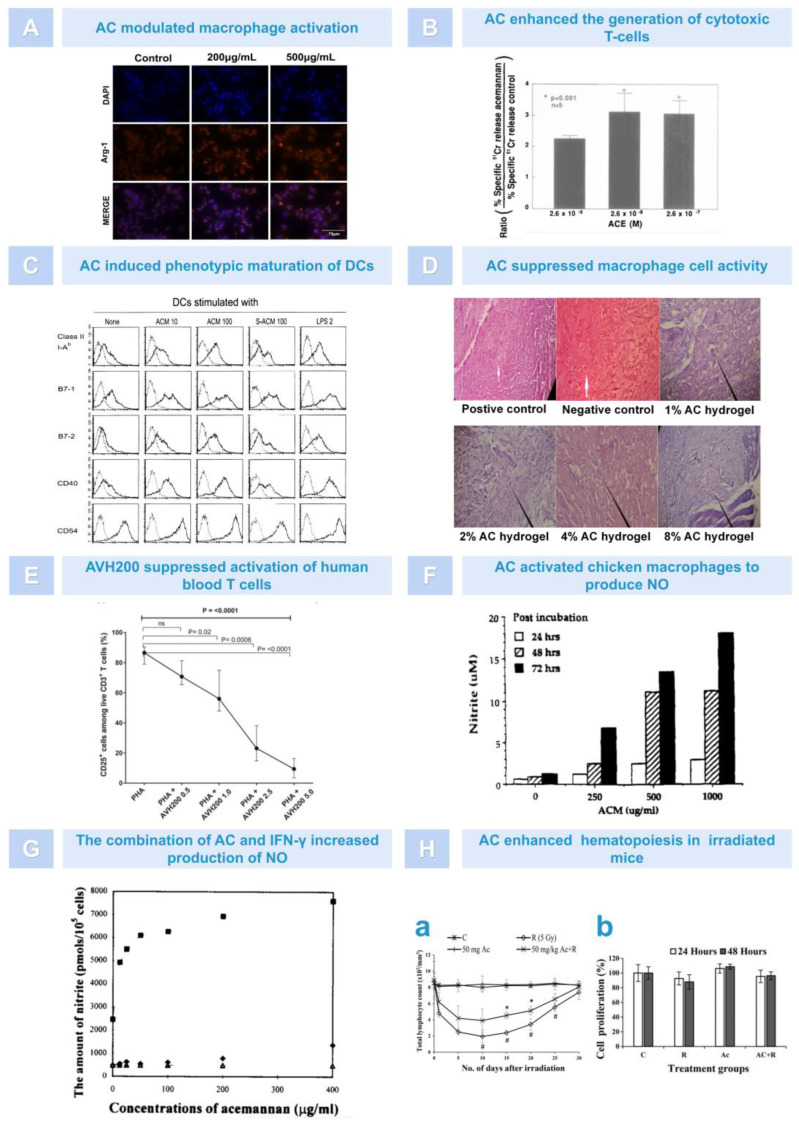
The immunomodulatory role of AC. (**A**). AC upregulated the expression of Arg−1, a marker of M2 macrophages [59]. (**B**). The effect of various doses of AC on generation of cytotoxic T lymphocytes [60]. (**C**). AC induced the phenotypic maturation of immature dendritic cells [61]. (**D**). Effects of AC on macrophage cells activity. Arrow: macrophages [62]. (**E**). AVH200 reduced the expression of CD25 among CD3+ T cells [63]. (**F**). AC enhanced NO production in a dose−dependent manner [65]. (**G**). NO were increased greatly in response to stimulation by a mixture of AC and IFN−γ. (−◆−: AC treatment alone. −■−: AC combined with IFN−γ.−△−: Medium alone) [66]. (**H**). AC promoted the activation of immune cells. AC enhanced peripheral lymphocytes count. # Compared with unirraiated control, *p* < 0.001; * Compared with irradiated control, *p* < 0.05 (**a**). AC enhanced spleen cell proliferation (**b**) [70].

Numerous studies have found that AC exerts good immune regulatory effects, mainly in the following three respects:

Firstly, as an immune enhancer, AC is typically used to activate one or more immune cells, thereby enhancing the body’s non-specific and specific immune functions. According to research findings, AC could regulate macrophage activation by targeting the PI3K/Akt/GSK-3β signaling pathway, enhancing macrophage M2 polarization and phagocytosis in RAW264.7 cells, thereby altering the phenotypic balance of macrophages in lung tissue and inhibiting lipopolysaccharide (LPS)-induced M1 polarization, ultimately enhancing the immune effect of the organism (Figure 4A) [59]. Moreover, AC could stimulate cytotoxic T cells (TCs) in a dose-dependent manner, promoting TC proliferation and enhancing TC cytotoxicity (Figure 4B) [60]. In addition to the two types of immune cells mentioned above, Lee et al. found that dendritic cells (DCs) treated with AC showed an increase in the expression of MHC-II, B7-1, B7-2, CD40, and CD54. Moreover, AC could enhance homologous mixed lymphocyte response, ultimately enhancing the immune activity of immature DCs (Figure 4C) [61]. However, some scholars proved that aloe gel, which is rich in AC, also had an immunosuppressive effect. For example, through in vivo experiments, Muharraran et al. found that AC hydrogel inhibited the activity of macrophages in a dose-dependent manner, thereby promoting the healing of tooth extraction wounds in rats (Figure 4D) [62]. Ahluwalia et al. found that A. vera inner leaf gel (AVH200), which is rich in AC, inhibited TC proliferation in a dose-dependent manner and led to a decrease in CD25 expression in CD3+T cells, as well as a decrease in IL-2, IFN-γ, and IL-17A secretion (Figure 4E) [63]. These results indicate that AC can exert different immune effects by regulating the activity of macrophages, lymphocytes, and dendritic cells.

Secondly, AC can also exhibit an immune character by promoting the generation of nitric oxide (NO). NO is synthesized by many cells involved in immunity and inflammation and is an important toxic defense molecule against infectious organisms. It also regulates the functional activity, growth, and death of a variety of immune and inflammatory cells, including macrophages, T lymphocytes, antigen-presenting cells, mast cells, neutrophils, and natural killer cells [64]. It was found that AC may induce NO synthesis in chicken macrophages through the terminal mannose receptor (Figure 4F) [65]. In addition, in the presence of interferon-γ (INF-γ), AC could significantly enhance the activation ability of macrophages, promote RAW 264.7 cell deformation, and increase surface antigen expression, especially the generation of NO (Figure 4G) [66,67]. These findings also provided a basis for better understanding the auxiliary activity of AC in viral and tumor diseases.

Thirdly, the immunoregulation effect of AC may also be reflected by increasing the effect of hematopoiesis. When AC was subcutaneously injected into mice, whose bone marrow was damaged after 7 Gy radiation, it enhanced the cellular nature of the spleen and peripheral blood, and the number of hematopoietic progenitor cells in the spleen and bone marrow increased. Furthermore, the enhanced hematopoietic activity of AC appeared to be stronger than that of the granulocyte colony-stimulating factor [68,69]. In addition, as above, after 7 days of pretreatment or post-treatment with AC, the survival rate of mice was significantly improved, which could be attributed to the upregulation of hematopoietic function (peripheral blood lymphocyte count, spleen cell number, spleen index), tumor necrosis factor-α (TNF-α), and interleukin-1 (IL-1) induced by AC in mice. These data suggest that AC had good immunomodulatory activity, protected against radiation-induced death in mice, and could be developed as a radiation damage mitigation agent (Figure 4H) [70].

### 5.2. Antiviral Effect

Aloe compounds have the potential to be used as antiviral drugs and immunomodulators to treat viral diseases, especially the use of emodin and AC in aloe as lead compounds [71]. Infection with human immunodeficiency virus (HIV) may cause organ failure and tumor development throughout the body, which is a serious threat to life and health. The consistent treatment of HIV is a challenge in the medical field. It is worth mentioning that, in several in vivo and in vitro studies, AC has been shown to help treat the immunosuppressive symptoms of immunodeficiency virus infection. For example, Kahlon et al. found that AC had a concentration-dependent inhibitory effect on the replication of HIV in CEM-SS cells infected with HIV-1 RFII strain. After treating the infected cells with AC, the viral load, free virus, syncytium formation, and cytopathic effect were all reduced [72]. Some scholars believed that the antiviral effect of AC was due to changes in the processing of oligosaccharide, which inhibited virus replication. Yates found that the intravenous or subcutaneous administration of AC in cats infected with feline immunodeficiency virus (FIV) improved survival rates, and a significant increase in lymphocyte counts was observed by blood analysis. This was important because a decrease in lymphocytes was the most common hematological manifestation of FIV infection in cats. Therefore, an increase in the lymphocyte count indicated that AC can help treat immune suppression caused by FIV infection [73]. The research also found that feline leukemia virus was also a retrovirus. AC had an impact on feline leukemia virus, which can significantly improve clinical conditions and survival rate [74].

**Figure 5:**
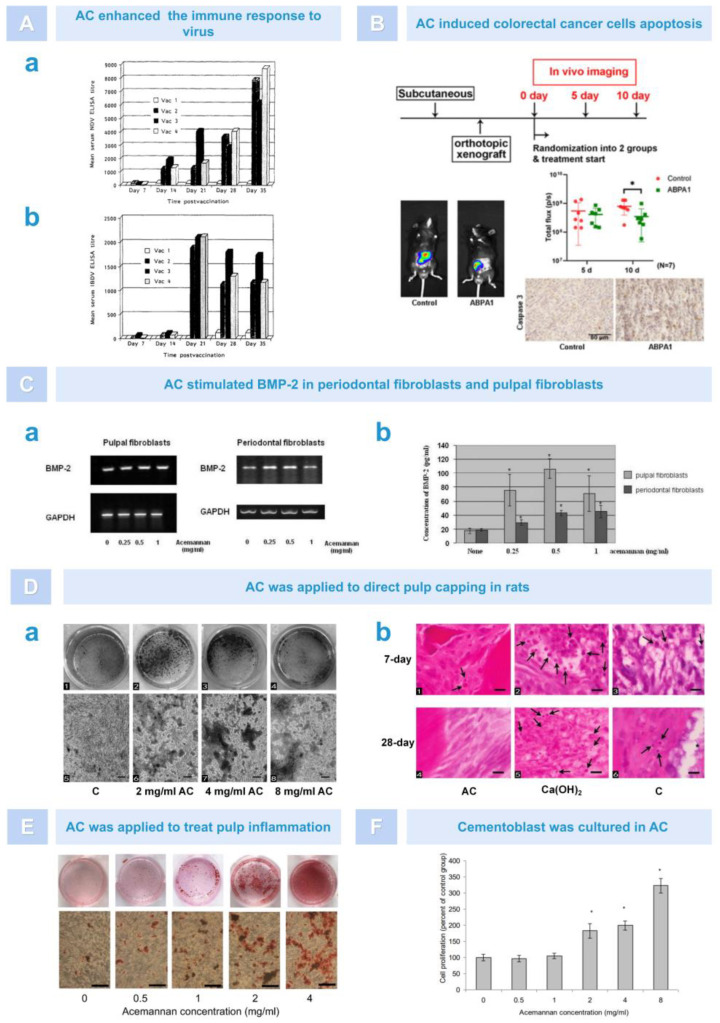
AC exerted antiviral and anti-tumor functions (**A**,**B**) and promoted dental tissue regeneration in cell and animal experiments (**C**–**F**). (**A**). AC enhanced the titer of protective antibodies. Comparison of the adjuvant effects of AC on the immune response to NDV (**a**) and IBDV (**b**) at 7 day intervals after vaccination. Vac l: saline, Vac 2: oil-based vaccine, Vac 3: vaccine-ACE-M, Vac4: vaccine and ACE m separately [77]. (**B**). AC was used in a mouse colorectal cancer model. Tumor imaging results showed that the volume of tumor tissue was reduced in the AC group, while immunohistochemical results showed that the expression of caspase-3 protein was increased. * Compared with the control group; *p* < 0.05 [81]. (**C**). Effect of AC on BMP-2 in pulpal fibroblasts and periodontal fibroblasts in an in vitro study. AC upregulated the expression of BMP-2 mRNA (**a**). AC induced the synthesis of BMP-2 in primary periodontal fibroblasts and pulpal fibroblasts. * Compared with the control group; *p* < 0.05 (**b**) [84]. (**D**). AC induced dentin formation in rat models. AC induced PDPCs mineralization (**a**) and inhibited the inflammatory response. Black arrow: inflammatory cells (**b**) [85]. (**E**). AC induced mineral deposition by deciduous pulp cells [86]. (**F**). AC enhanced cementoblast proliferation. * Compared with the untreated group; *p* < 0.05, n = 3 [87].

In addition to directly acting on viruses, AC is also used in the preparation of adjuvants in viral vaccines. Adjuvants, also known as biological response modulators (BRM), are an additional vaccine component that can enhance the immune response, increase antibody titers, change the type of antibody production, and cause or enhance delayed hypersensitivity reactions [75]. Although many adjuvants are licensed for certain vaccines, there is still a need for safer substitutes that stimulate antiviral immune responses. In animal experiments, AC, as a BRM, significantly increased the anti-coxsackievirus B3 (CVB3) antibody titer produced during acute infection of three strains of mice with CVB3. Moreover, AC may increase the antibody titer of other enteroviruses during a natural infection and poliovirus vaccine strains [76]. In addition, as a vaccine adjuvant, AC could significantly enhance the protective antibody titer against Newcastle disease virus (NDV) and infectious bursal disease virus (IBDV) after vaccination in day-old broiler chicks, which indicates that AC has an antigen-dependent adjuvant property and can enhance the immune response to NDV and IBDV (Figure 5A) [77]. However, in a mouse model of myocarditis, AC did not achieve the desired antiviral effect, and the specific reason for this has not yet been investigated [76].

### 5.3. Anti-Tumor Effect

Currently, it is believed that AC exerts its antitumor activity by activating the multipotent effector cells in the immune system, mainly macrophages, thereby stimulating the production of cytokines [78]. For instance, AC can activate macrophages to produce monokines, such as IL-1 and TNF, which stimulate blastogenesis in thymocytes and induce necrosis and the disappearance of Norman mouse sarcoma [79]. In addition, studies have reported that AC can help treat colon cancer. Previous studies have found that, by reducing the activation of nuclear factor kappa B (NF-Κb), the processed *Aloe vera* gel (PAG), which is rich in AC, can inhibit inducible NO synthase and cyclooxygenase-2 expression. Moreover, PAG can induce the expression and phosphorylation of cytokines by reducing signal transducers, transcriptional activator 3, and cell cycle progression, thereby promoting apoptosis in colon cancer [80]. In a recent study, Tong et al. found that polymeric AC extracted from aloe can inhibit tumor growth in an orthotopic colon cancer model. AC altered mitochondrial membrane permeability by promoting Bax translocation while causing cytochrome-c release, which triggered the caspase cascade reaction. This elucidated the mechanism by which AC induced apoptosis in colorectal cancer cells and suggested that AC may have potential applications in the development of mitochondria-targeted anticancer drugs (Figure 5B) [81].

### 5.4. Dental Tissue Regeneration

Periodontal fibroblasts and pulpal fibroblasts are considered the main cells for the formation of new alveolar bone and dentin, respectively. Bone morphogenetic protein 2 (BMP-2) plays an important role in inducing new odontoblast formation and wound healing [82,83]. In 2007, Jittapiromsak et al. proposed that AC could stimulate the expression of BMP-2 in pulpal and periodontal fibroblasts, which laid the foundation for the theory that AC promoted hard tissue regeneration (Figure 5C) [84]. Subsequently, in many in vivo and in vitro experiments, AC has been shown to be useful for dental tissue regeneration engineering due to its ability to promote cell proliferation, cytokine expression, and tissue healing. For example, in animal models, Jittapiromsak et al. evaluated the regenerative effect of AC as a pulp capping agent on rat dentin, using calcium hydroxide as a positive control group. The results revealed that the AC-treated group exhibited intact and uniform calcified dentin bridges and good pulp tissue formation with no or only mild inflammation in the soft tissues. However, no complete dentin bridge formation was detected in both the calcium hydroxide-treated group or the sham-operated group, and there were varying degrees of inflammation in the soft tissue (Figure 5D) [85]. Subsequently, Songsiripradubboon et al. used AC in an animal model of pulp inflammation and observed that AC successfully induced dentin regeneration with an effect comparable to that of mineral trioxide aggregate (MTA). Furthermore, they found that the in vitro treatment of primary human dental pulp cells (PDPCs) with AC stimulated pulp cell proliferation and differentiation in adult dentin-like cells and promoted BMP-2, type I collagen (COL-1), and dentin salivary protein expression (Figure 5E) [86]. In addition, AC may also be a drug that could be used to promote the regeneration of cementum. In another in vitro experiment, it was found that AC significantly stimulated the proliferation and differentiation of cementoblasts, promoted the secretion of growth factor and the formation of extracellular matrix, and significantly induced mineral deposition on the experiment’s 21st day (Figure 5F) [87]. In summary, due to its good biocompatibility, AC promotes the formation of dental tissue by stimulating cell proliferation, differentiation, extracellular matrix formation, and mineralization.

### 5.5. Osteogenesis

**Figure 6:**
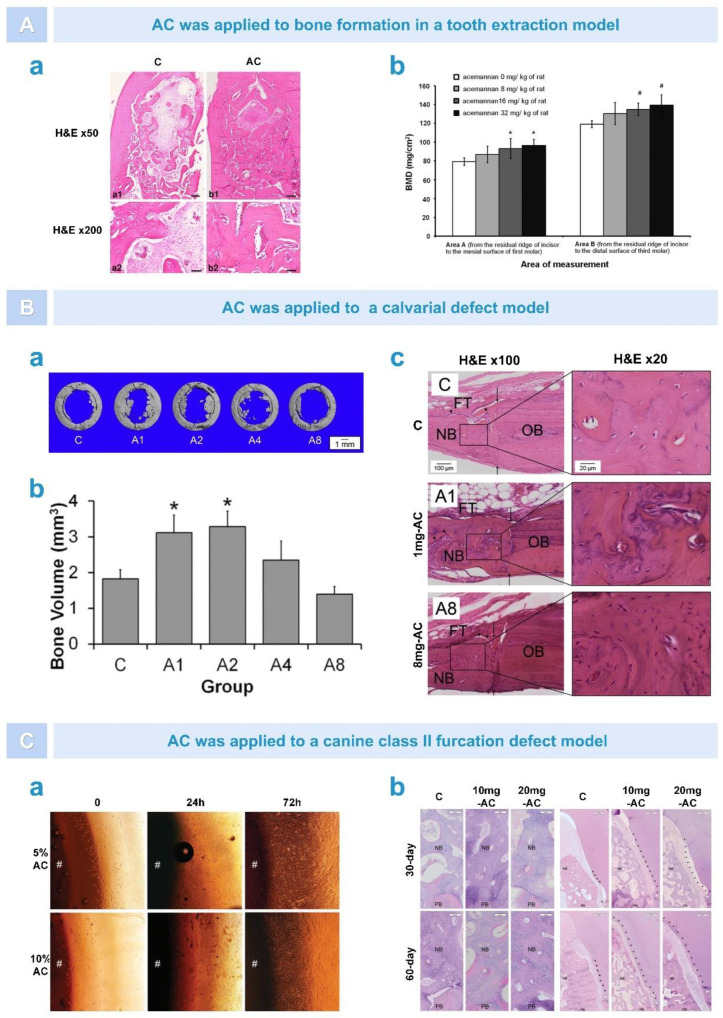
AC stimulated bone regeneration in different bone defect models. (**A**). AC induced bone formation and promoted tooth socket healing in a rat model. Compared with the control group, an increase in the thickness and density of the bone matrix of bone trabeculae were observed in the AC group (**a**). AC increased bone mineral density in tooth socket *,# Compared with the untreated socket; *p* < 0.05, n = 7 (**b**) [88]. (**B**). AC promoted calvarial defect healing. AC increased bone volume. * Compared with the blood clot control group, *p* < 0.05 (**a**,**b**). AC stimulated osteoblast maturation. A1: 1 mg AC; A2: AC 2 mg AC; A4: 4 mg AC; and A8: 8 mg AC; Black arrow: defect border; OB: old bone, NB: new bone, FT: fibrous tissue (**c**) [35]. (**C**). AC stimulated the regeneration of alveolar bone and periodontal tissue. AC had biocompatibility with periodontal ligament cells (**a**) and induced more new bone and cementum formation than the control group. NB: new bone; PB: pre-existing bone; black arrowhead: new cementum; white arrowhead: pre-existing cementum; white arrow: the apical limit of the defect (**b**) [38].

Experimental studies have shown that AC can stimulate the proliferation and differentiation of stem cells, improving the speed and quality of bone tissue regeneration. For instance, Boonyagul et al. co-cultured AC with bone marrow mesenchymal stem cells (BMSCs) or PDLCs in vitro and found that AC dose-dependently stimulated the proliferation and differentiation of BMSCs and PDLCs and upregulated the expression of various cytokines such as runt-related transcription factor 2 (Runx2) and growth differentiation factor 5 (GDF-5) and BMP2, ultimately promoting alkaline phosphatase activity and mineral deposition [38,88]. AC has also shown the ability to promote bone regeneration in various animal models of bone defects. For example, in a rat mandibular incisor extraction model, a sponge treated with AC was placed in the alveolar fossa. Postoperative imaging results showed that bone density increased and bone healing accelerated in the extraction fossa. Histologically, a large number of bone trabeculae was observed (Figure 6A) [88]. In a rat skull defect model, imaging observations showed that the addition of AC significantly increased the surface area, volume, and tissue mineral density of newly formed bone. Histological observation showed that the bone matrix was denser than the control group and that cubic bone-forming cells were formed in the active bone formation area around the newly formed bone (Figure 6B) [35]. In a canine class II furcation defect model of dog premolars, after 30 days of treatment with AC, histological analysis showed that the defect was filled with new alveolar bone, and tooth cement and periodontal ligament were formed (Figure 6C) [38]. In summary, AC is an effective bone regeneration bioactive agent that can be used to repair various types of bone defects.

### 5.6. Soft Tissue Healing

**Figure 7:**
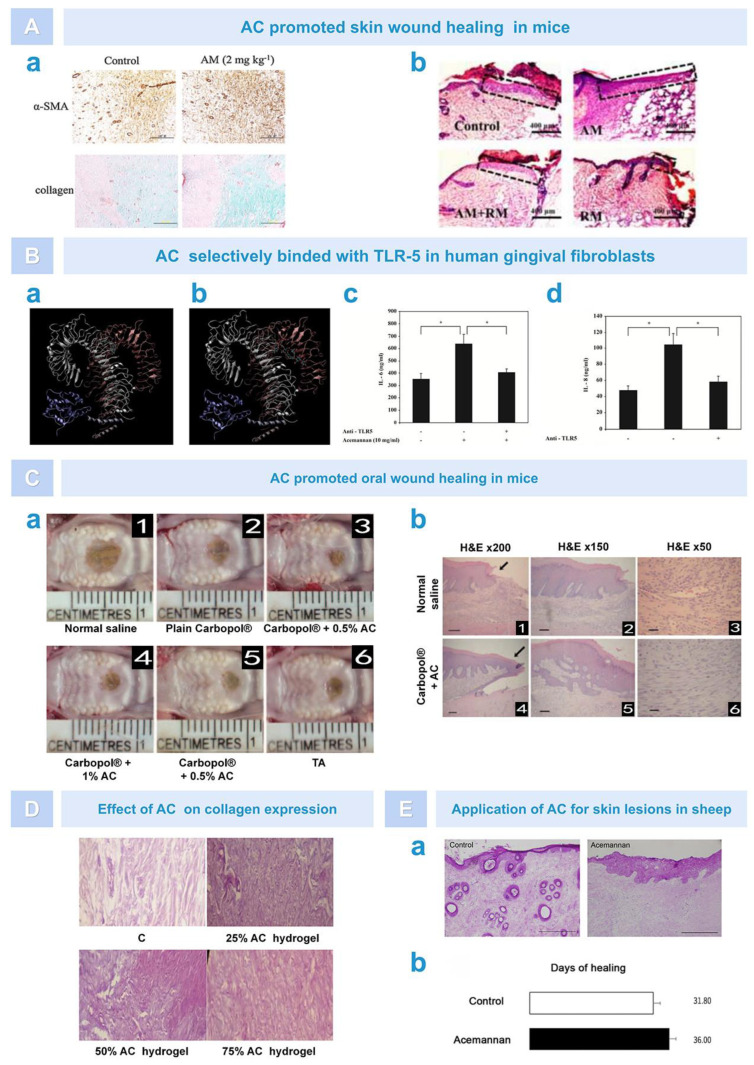
Application of AC in soft tissue healing. (**A**). Effect of AC on skin wounds. Immunochemistry staining showed that AC stimulated fibroblasts and collagen secretion (**a**) and enhanced the re−epithelization of the wound area. Dotted box: epithelial migrating tongue. (**b**) [89]. (**B**). The binding energies between AC and TLR5. Monomeric- (**a**) and dimeric- (**b**) single-stranded AC molecules bound to the Htlr5 ectodomain with different binding energies. The use of anti−TLR5 antibodies significantly reduced AC−induced IL−6 (**c**) and IL−8 (**d**) secretion. * Between the groups; *p* < 0.05 [90]. (**C**). AC was applied to oral wound healing. AC significantly promoted oral wound closure (**a**) and showed better results in the histopathological images compared with the control group. Black arrow: re-epithelization front (**b**) [91]. (**D**). AC increased collagen density in the gingival tissue in a diabetes mellitus animal model [40]. (**E**). Effects of AC on skin healing. Histopathology showed that immature granulation tissue was seen in AC−treated wounds (**a**), and AC prolonged the days of healing compared with the control group (**b**) [92].

**Figure 8:**
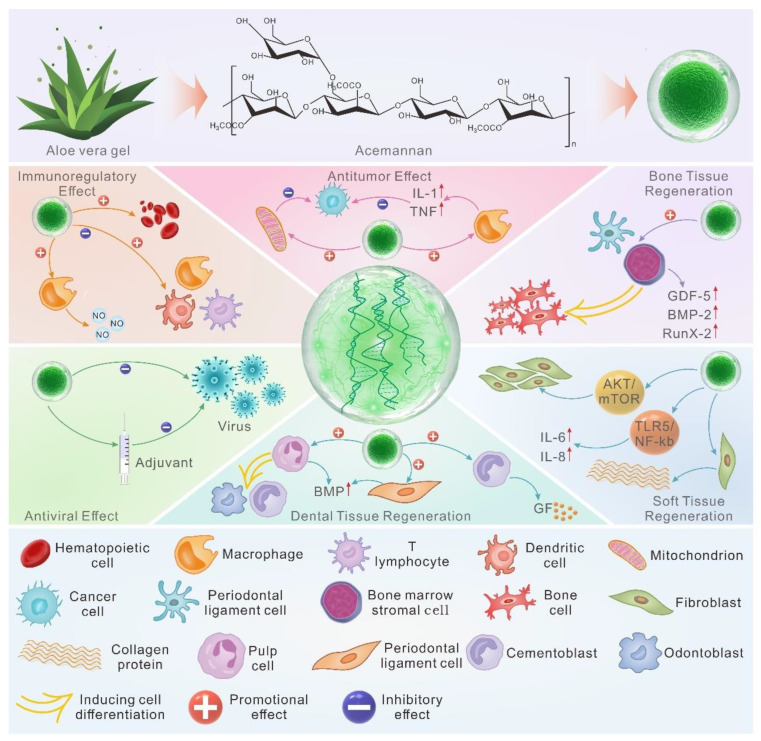
Function and mechanism of AC in cell and animal experiments. AC can exert different biological functions by stimulating cell proliferation and/or cytokine expression, such as immunomodulatory effects, antiviral effects, anti-tumor effects, dental tissue regeneration, bone tissue regeneration, and soft tissue regeneration.

AC can promote soft tissue healing by stimulating fibroblast proliferation and cytokine expression. Xing et al. found that AC promoted the expression of fine cyclin D1 in fibroblasts through the AKT/mTOR signaling pathway, enhanced activity of eukaryotic translation initiation factor-4F (eIF4F) in eukaryotic cells, and increased the translation of cyclin D2 to promote skin wound healing (Figure 7A) [89]. Thunyakitpisal et al. found that monomer/dimer single-chain AC molecules had a high binding affinity with TLR5 flagellin recognition sites. Through the TLR5/NF-ĸb-dependent signaling pathway, AC can induce the expression of IL-6/-8 and p50/DNA binding in gingival fibroblasts (Figure 7B) [90]. In other animal models, AC also plays a role in promoting tissue healing. For example, Jettanacheawchankit et al. placed AC on hard palate wounds in rats. After 7 days, they found that the wound healing effects were significant, and the 0.5% concentration of AC had a better curative effect than triamcinolone acetonide. Histological analysis found that the wound edge of the AC-treated group was covered by epithelial cells, and the connective tissue layer showed relatively fewer inflammatory cells and more fibroblasts (Figure 7C) [91]. Muharlaran et al. extracted 30 rat teeth and put 1%, 2%, 4%, and 8% AC gel into the alveolar fossa. The results showed that AC gel could promote wound healing after tooth extraction by inhibiting macrophage activity, and 8% AC had the strongest inhibitory activity [62]. Susanto et al. explored the potential of AC to repair the gingival tissue in diabetic rat models and conducted an in vivo experimental study on 24 diabetic rats divided into four groups (negative control, 25%, 50%, and 75% AC hydrogel). The results showed that the collagen density fraction of rats’ gingiva increased with the increase in AC gel concentration, which indicated that AC can repair gingival tissue by improving the collagen density of gingival tissue in diabetic animal models (Figure 7D) [40]. Iacopetti et al. applied AC gel to the back wound of sheep and found that AC dehydrated wounds and stimulated the proliferation of granulation tissue and cells; therefore, AC had a positive effect on granulation tissue and secretion-rich moist wounds. However, AC could not reduce the closure time of the wound and produced a certain degree of inflammatory response (Figure 7E) [92]. The function and mechanism of AC in cells and animals are shown in Figure 8 and Table 1.

## 6. Advances in the Clinical Application of AC

### 6.1. Regeneration of Dental Pulp–Dentin Complex

**Figure 9:**
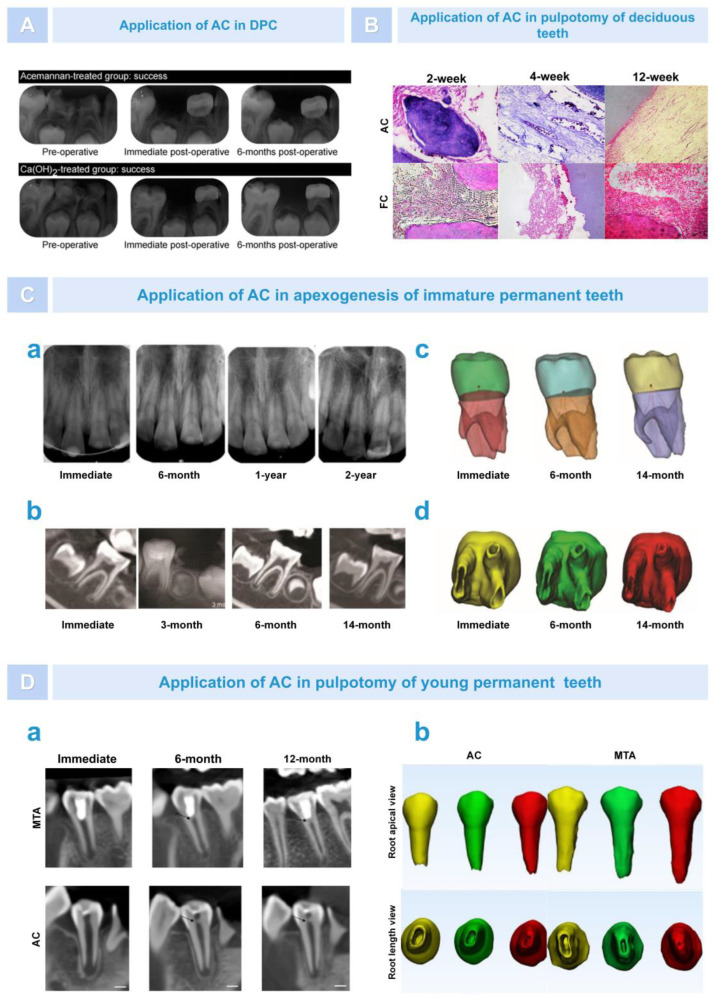
The effect of AC on the regeneration of the pulp–dentin complex. (**A**). Radiograph of AC group and control group [36]. (**B**). Histopathological images of AC group and FC group [98]. (**C**). Role of AC in apexogenesis of immature permanent teeth. Radiographs (**a**,**b**) and 3D reconstruction images (**c**,**d**) showed that AC successfully promoted root development and reduced the apical foramen area in young permanent teeth [100]. (**D**). Role of AC in pulpotomy of young permanent teeth. According to X-ray images (**a**) and 3D reconstruction images (**b**), AC induced continued root formation in immature permanent teeth. Black arrow: dentine bridge; yellow: immediate; green: 6 months; red: 12 months [99].

For teeth with exposed pulp caused by mechanical or traumatic factors, in order to avoid newly stimulating the pulp, it is necessary to use direct pulp capping (DPC) to cover the pulp’s exposed area to promote pulp healing and preserve pulp vitality [93]. Excellent pulp capping agents should have good biocompatibility, be non-irritating and non-toxic to dental pulp, and have a strong ability to sterilize and promote the repair and regeneration of dental pulp tissue. At present, the commonly used pulp capping agents in clinical practice include calcium hydroxide and MTA. However, they also have some shortcomings. For example, calcium hydroxide does not have inherent adhesive properties and is more irritating to dental pulp [94,95]. MTA has the problems of a long solidification time and high cost [96]. Therefore, identifying an excellent biomaterial for use as a capping agent has been a problem that scholars have been exploring. In previous animal models, there have been numerous reports on the use of AC as a pulp capping agent to promote the formation of restorative dentin [85,86]. In clinical treatment, in 2015, Songsiripradubboon et al. evaluated the effect of AC as a DPC agent on the treatment of deep caries in deciduous teeth compared to calcium hydroxide. The research team immediately disinfected the deep caries molars of 37 children after removing the decay and exposing the pulp and randomly placed AC or calcium hydroxide. After six months, there was no significant difference in the overall clinical and imaging success rates (72.73% and 70.0%, respectively) of direct pulp capping with AC and calcium hydroxide. However, compared with the calcium hydroxide control group, the histopathological results of the AC experimental group showed that pulp inflammation was milder, the formation of a dentin-like cell layer could be seen near the dentin bridge, and the generated pulp tissue was very similar to healthy pulp tissue (Figure 9A) [36]. In addition, research has found that AC can be used as a denture adhesive. A 150:1 denture adhesive formula is the most ideal in terms of strength, pH value, and cytotoxicity. Therefore, in terms of bonding performance, using AC as a pulp capping agent may compensate for the weak adhesive property of calcium hydroxide [10].

In addition to DPC, pulpotomy is an important treatment method for preserving the pulp and is often considered the preferred method of treating exposed pulp tissue and promoting root growth [97]. In the past two years, scholars from various countries have also innovatively applied AC in pulpotomy. Gonna et al. used AC and formocresol (FC) as pulp capping agents to compare their effect in terms of pulpotomy for primary teeth. The results showed that AC had more advantages as a pulp cutting agent than FC. AC has a stronger ability to induce dentin formation and no obvious signs of pulp inflammation, providing a valuable natural alternative biomaterial for the endodontic treatment of baby teeth (Figure 9B) [98]. In addition, Vu et al. compared the application of AC with MTA in the pulpotomy of young permanent teeth using a 3D Tooth Reconstruction model. The results showed that, through 0–12 months of follow-up, the root length of both groups increased, and the apical area decreased significantly. There was no significant difference in the total success rates, which were 90.91% (AC group) and 95.65% (MTA group) (Figure 9D) [99]. In 2022, Vu et al. published a report on two cases of AC being used for pulpotomy. After 12 months, the results showed that the young permanent teeth exposed to two dental pulps after treatment with AC were asymptomatic, the pulp remained viable, and apical foramen were successfully induced. In addition, the oxygen level (oxygen saturation) in the pulpal blood supply in the AC group was tested via pulse oximetry within a normal range (Figure 9C) [100]. These experiments demonstrated that the use of AC as a capping material preserved pulp vitality, simultaneously induced root formation, and had the same tendency as MTA in the treatment results of partial pulpotomy, with lower costs compared to MTA.

### 6.2. Bone Regeneration 

#### 6.2.1. Osteogenesis after Alveolar Surgery

**Figure 10:**
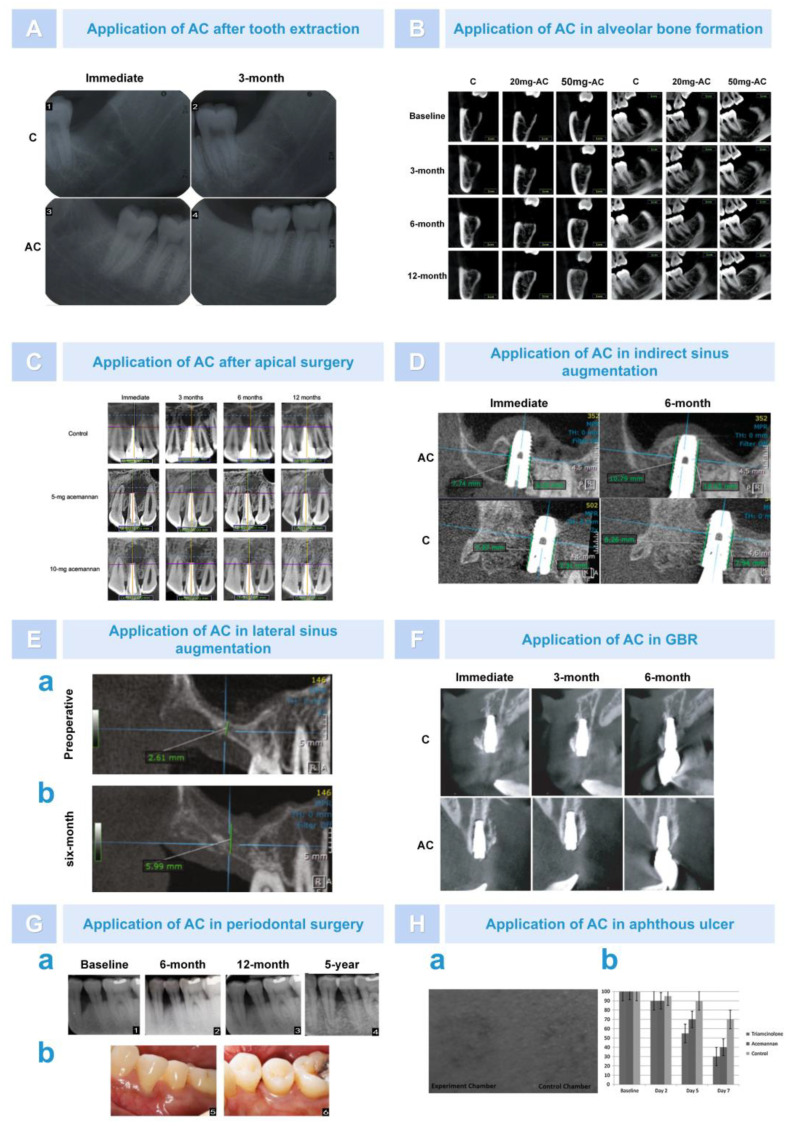
The effect of AC on bone regeneration. (**A**). AC reduced tooth socket volume after tooth extraction [37]. (**B**). CBCT showed that AC promoted alveolar bone formation after tooth extraction [101]. (**C**). AC induced rapid early osseous defect healing after apical surgery [102]. (**D**). AC enhanced endo-sinus bone formation greatly in indirect sinus augmentation [34]. (**E**). AC significantly increased bone height in lateral sinus augmentation surgery. The CBCT images of sagittal view at preoperation (**a**) and six months postoperation (**b**) [105]. (**F**). AC enhanced the dimensional stability of the regenerated tissue [107]. (**G**). AC induced periodontal tissue regeneration during periodontal surgery. AC increased radiographic bone fill (**a**) and improved the clinical appearance of the treated tooth (**b**) [110]. (**H**). AC was effective in the treatment of an aphthous ulcer. AC reduced ulcer size (**a**,**b**) [111].

In rat tooth extraction models, AC has been shown to serve as a bioactive molecule that can induce alveolar bone formation by stimulating BMSC proliferation, differentiation into osteoblasts, and extracellular matrix synthesis [88]. In a recent clinical experiment, Jansisyanont et al. investigated the effect of AC on alveolar bone healing after mandibular third impact molar extraction surgery. After extraction, 99 cases of mandibular third impact molars were randomly placed into AC sponges. Three months later, it was found that the AC-treated group significantly increased the rate of bone tissue formation in the alveolar fossa, and bone radiographic density increased by approximately 15% to 17% (Figure 10A) [37]. However, the disadvantage of this experiment was that the observation period was short, and it was based on two-dimensional rather than three-dimensional images. Subsequently, Vu et al. analyzed the long-term efficacy of different doses of AC on alveolar healing after tooth extraction using cone-beam computed tomography (CBCT). After 12 months, the results showed that AC significantly reduced the volume of the alveolar fossa in all groups, and the effect of 50 mg AC was more obvious. Through the use of electron microscopy, bone trabecular formation and increased interconnectedness were observed, and thick layers of dense bone formation were visible at the alveolar ridges (Figure 10B) [101]. In addition, Le Van et al. applied AC in the study of bone healing during apical surgery. It was found that, at 3 months after surgery, the osteogenesis rate in the AC group had a faster osteogenic rate in the surgical area, and the percentage of total bone defect volume reduction (%ΔBDV) was significantly increased (Figure 10C) [102]. Moreover, in clinical practice, AC in the form of SaliCept patches has been found to reduce the incidence of common complications such as alveolar osteitis in tooth extraction and was more effective than clindamycin-soaked gelatin sponges [103]. These experiments demonstrated that AC, as a biomaterial, could promote alveolar bone healing through its potential osteoinductive activity.

#### 6.2.2. Bone Augmentation in Oral Implantation Area

Insufficient bone mass is one of the key issues affecting implant surgery. On the one hand, when bone is deficient in the posterior maxillary region, bone graft materials are usually inserted after lifting the maxillary sinus floor elevation to increase bone mass [104]. AC has been proved to be a bone graft material in previous experiments. Trinh et al. used AC as a bone graft material for the maxillary sinus floor elevation to study its clinical osteogenic performance. Thirty patients undergoing indirect sinus augmentation surgery with the simultaneous implantation of implants were randomly divided into two groups: a blood clot control group and an AC sponge transplantation group. At 3 and 6 months after surgery, CBCT results showed that the percentage endo-sinus bone gain in the AC-treated group was approximately 2.4 times and 2 times higher than that in the control group (Figure 10D) [34]. In addition, there has been some progress in the osteogenesis effects of AC during lateral sinus augmentation surgery. Trinh et al. reported the case of a 57-year-old female patient who underwent a two-stage maxillary sinus lift using AC sponges. Six months after surgery, it was found that the height of the alveolar bone increased significantly from 2.61 mm to 5.99 mm. Histological analysis revealed that the AC sponges were absorbed and replaced by neonatal bone, and osteoblast-rich bone, calcified areas, and thick bone trabeculae were seen in the sinus (Figure 10E) [105]. This indicated that AC had great degradability, bone induction, and biocompatibility. The author concluded that the osteogenic effect of AC was superior to that of the blood clot group, mainly because the early rupture of the blood clot during the healing process led to membrane collapse and limited the amount of new bone. In contrast, AC sponge treatment provided a highly plastic bone conduction scaffold and space maintenance, helping to avoid damaging the sinus membrane while maintaining the volume of the supporting sinus membrane to promote bone tissue regeneration. 

On the other hand, when the horizontal and vertical bone mass of the alveolar ridge was insufficient, severe bone defects in the edentulous area cannot guarantee good initial stability and the ideal three-dimensional position of the implant. The use of guided bone regeneration (GBR) can enable patients to obtain a certain amount of bone increment in the implant area [106]. Deesricharoenkiat et al. used AC and deproteinized bovine bone (DBB) together for clinical cases of maxilla defects. After 3 months of implantation, they found that the height of the alveolar bone defect was significantly reduced at each measured position. This indicated that AC had osteogenic properties and had potentially beneficial effects on implant stability in the early stages of healing. However, the effect of osteogenesis was not significant at 6 months (Figure 10F) [107]. In addition, studies found that AC sponge treatment can affect the implant stability quotient (ISQ) of implants by enhancing bone bonding between implants and bone. However, the difference in the results was not significant. This suggests that the dosage and application form of AC need to be further explored.

#### 6.2.3. Repair of Periodontal Tissue after Treatment of Periodontitis

In the treatment of periodontal disease, AC can also play a significant role and be used for the repair of periodontal tissue. Lpshita et al. compared the effects of *Aloe vera* (AV) gel (mainly composed of AC) and alendronate sodium (ALN) gel as local immunomodulators after periodontal scaling and root planing. Compared with the ALN group, at 6 and 12 months after surgery, clinical and imaging data showed that the depth of bone defects in the AV group was not significantly reduced but significantly improved compared to the placebo group. Furthermore, clinical indicators such as periodontal pocket depth, attachment loss, and modified gingival sulcus bleeding index (mSBI) all decreased [108]. Moreover, the test group using AV gel experienced significant improvements regarding the mSBI and periodontal index, which can be attributed to its anti-inflammatory and immunomodulatory effects. AV gel can downregulate the expression of inflammatory cytokines induced by LPS, accelerate collagen synthesis, and promote wound healing [109]. In a recent experiment, Chansamart et al. reported a case whereby AC sponge treatment was deployed to help three patients with severe chronic periodontitis and significant bone defects after periodontal surgery; the follow-up period was set to 5 years. The results showed that the clinical outcomes were better compared to using open flap debridement alone. The clinical indicators, such as probing depth and periodontal attachment level, were improved, and radiographic bone filling was increased (Figure 10G) [110]. Moreover, in order to fully demonstrate the regeneration effect of AC in periodontal tissue, this research team is currently conducting a study using AC for guided tissue regeneration (GTR). In summary, AC may be a useful auxiliary plant-derived biomaterial for periodontal tissue regeneration.

### 6.3. Treatment of Skin and Mucosal Disease

AC has a good effect in the treatment of oral ulcers. Previous studies have shown that AC can increase the proliferation of epithelial cells and fibroblasts by activating growth factors. Animal experiments have also demonstrated that AC can accelerate oral wound healing and regulate immune activity [91]. It has been reported that AC hydrogel can accelerate the healing of aphthous ulcers and reduce pain [112]. Bhalang et al. found that the use of AC in the treatment of oral aphthous ulcers reduced the ulcer area to the same extent as 0.1% triamcinolone acetonide by the next day. Although, on the fifth and seventh days, AC reduced the ulcer area slightly less than triamcinolone; however, the curative effect was better compared to the blank control group. In addition, although the efficacy of AC cannot be comparable to 0.1% triamcinolone acetonide, AC derived from natural plants is a good option for patients avoiding steroid hormones (Figure 10H) [111]. AC also has a good effect on skin wound healing. In 1998, Chithra et al. found that topical and oral treatment with *Aloe vera* had a positive effect on glycosaminoglycan (GAG) synthesis, thereby beneficially regulating skin wound healing [113]. Thomas evaluated the efficacy of AC dressings on pressure ulcers and compared them with wet saline gauze dressings. During the 10 week observation period, 3 out of 30 subjects (10%) had their ulcers completely healed, and no difference was observed between the experimental group and the control groups in terms of complete healing. This study showed that, in the treatment of pressure ulcers, AC hydrogel dressing was as effective as wet saline gauze wound dressing but not superior to it [114]. In addition, it found that *Aloe vera* gel, which is rich in AC, can be used as an adjuvant to promote connective tissue repair, treat dermatologic microscopic polyangiitis, and promote the vascularization of traumatic surfaces.

### 6.4. Reduction in Blood Sugar and Blood Lipids

**Figure 11:**
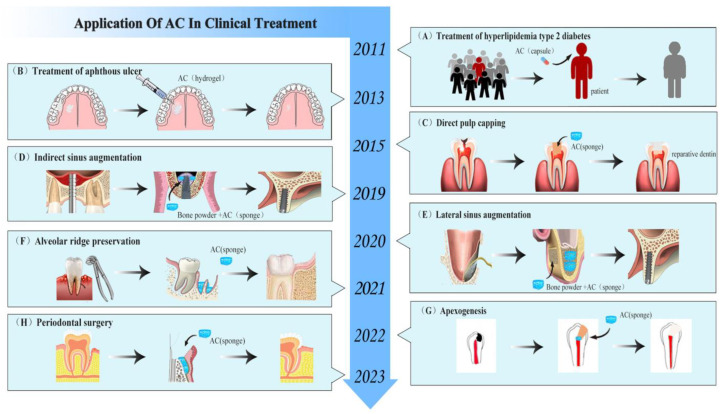
Advanced clinical application of AC.

*Aloe vera* has a variety of pharmacological effects and has been found to lower blood glucose and blood lipids, which is associated with many of its active ingredients. For example, the polysaccharide component can regulate glucose metabolism by activating gluconeogenesis and inhibiting gluconeogenesis, and phytosterols can reduce serum-free fatty acids and triglyceride levels and improve glucose homeostasis and lipid metabolism [115]. Huseini et al. administered 300 mg of *Aloe vera* gel capsules (AC) orally every 12 h to 35 patients with type 2 diabetes to assess the effectiveness of *Aloe vera* gel in lowering blood sugar. The results showed that, compared with the placebo group, aloe leaf gel significantly reduced the level of fasting blood glucose and glycosylated hemoglobin and had no significant impact on blood lipids and liver and kidney function tests [116]. Then, they administered the *Aloe vera* gel orally to 33 type 2 diabetic patients with high blood lipids to evaluate its effect on blood lipids. In alignment with previous results, *Aloe vera* gel not only significantly reduced fasting blood glucose and glycated hemoglobin but also reduced total cholesterol and low-density lipoprotein levels, which indicates that AC may have a positive effect on lowering blood glucose and lipids [117]. However, there is little research on the biological activity of AC and its mediated anti-hyperglycemic mechanisms, so further clinical trials are seemingly necessary. The advanced clinical application of AC in recent years is shown in Figure 11 and Table 2. 

## 7. Combined Application of AC and Other Compounds

AC has been shown to have good immune regulation, tissue regeneration, and antitumor and antiviral activities. In clinical treatment, AC can promote the repair of alveolar bone, skin, oral mucosa, and dentin. However, there are still many limitations to individual applications. For example, encapsulating AC for oral administration cannot control the release rate of drugs in the body. AC can be considered to combine high molecular compound carriers to create a certain dosage form to control the dissolution rate of the drug in the body so that AC can be slowly released at a certain speed within the required time range according to the designed dosage. Because the gel is in liquid form and has large fluidity, when AC in the form of gel is applied to repair epidermis and mucous membrane, its efficacy is easily affected by body position and application site, and it can be considered for preparing finished dressing by combining AC with other compounds for clinical use. If the lesion occurs in load-bearing areas such as articular cartilage, the use of spongy AC alone cannot effectively recover the defect. It is often necessary to combine other drugs to prepare materials with excellent resistance and retention performance. AC can also be used in combination with other compounds to compensate for the shortcomings of other compounds in terms of activity, structural characteristics, and physiological functions. The purpose of this section is to introduce the properties of new materials formed by AC and other compounds and discuss the possibility of their combined application in order to provide a scientific basis for future research into the clinical use of AC.

### 7.1. Combining AC with Polysaccharide

#### 7.1.1. Chitosan (CS)

CS is a deacetylated natural biopolymer derived from chitin. CS biomaterials have unique properties, such as biocompatibility, non-toxicity, mucosal adhesion, and extensive antibacterial and antifungal activities [5]. AC and CS have been used in combination in many fields. Firstly, the combination of AC and CS can help in designing new composite materials for applications in fields such as medicine, industry, and transportation. Although the physical properties and biological activities of the AC/CS mixed hydrogel formed by the chemical cross-linking method and the AC/CS film formed by the solution casting method were not ideal in experimental tests, the emergence of these composites provided hope for the development of biomaterials such as scaffolds for wound treatment in the future [41,118]. Following subsequent research, it was found that the AC/CS aerogel material obtained by drying the physical hydrogel formed by the two through supercritical CO_2_ treatment verified the potential of their combined application for wound recovery. Because of its low density and high porous structure with a high specific surface area, the AC/CS aerogel material can provide a higher liquid absorption rate and enhance the interactions in living tissue, thus achieving faster and more effective wound healing. In addition, the combined application of AC/CS in bone tissue formation has also been confirmed. In vitro experiments have found that adding AC to CS coatings can inhibit the release of osteoblasts and increase their density and activity. In a rat femoral model, it was found that doping AC and CS on a hydroxyapatite coating covering titanium implants can improve the degree of bone mineralization and accelerate the speed of bone healing, thus greatly improving the osseointegration of the implant [119]. Secondly, the combination of AC and CS also has outstanding advantages regarding drug delivery. Lipid nanoparticles modified with AC and CS can serve as a promising liposoluble antibiotic intracellular transport carrier. Through physical stability testing, it was found that the particle size and content of AC-CS lipid nanoparticles remained highly stable. Cell viability experiments using Vero cells and BALB/c 3T3 cells revealed that the nanoparticles were not toxic and showed the cellular uptake of rifampicin loaded AC/CS lipid nanoparticles in BALB/c 3T3 cells [120]. Thirdly, the combination of the composite scaffold material and seed cells is applied in tissue regeneration engineering. Scaffold materials and seed cells are essential elements for tissue regeneration engineering. Combining AC/CS composite scaffolds with periodontal ligament-derived stem cells had the potential to treat tissue defects and restore bone defects after ameloblastoma resection due to its excellent inherent antibacterial properties, mechanical properties, and biocompatibility [121].

#### 7.1.2. Alginate (ALG)

ALG is an edible heteropolysaccharide that widely exists in brown algae and bacterial capsules. Due to its gel forming characteristics, it is widely used in food, textile, paper, and other industries. However, its greater fluidity also causes ALG to form a soft structure when it makes contact with the physiological environment, which limits its potential in soft tissue regeneration. In order to solve this problem, AC is added to ALG to make a solid mixed material [122]. It was found that the membrane structure formed by AC and ALG had higher mechanical strength and stability. This was because the Ca^2+^ in AC increased the network stability favored by the physical interaction with ALG and promoted its gelation and formation of egg-box conformation. Moreover, in the food industry, the application of AC and ALG coatings can increase the ascorbic acid content and antioxidant potential of fruits and preserve their sensory, nutritional, and functional properties, providing new ideas for their future use in the fields of medicine and cosmetics [123]. In addition, it was found that AC was combined with CS and ALG via the use of electrospinning technology to build a multicomponent system. Through different intermolecular interactions, a complex ternary polyelectrolyte membrane structure was formed. Compared with the composite binary compound of AC, it had a more stable network structure and stronger interaction between polymer chains, which enabled the film to have better dimensional stability, flexibility, and expansion ability (in terms of physical properties). Moreover, this blended film can promote the attachment, diffusion, and vitality of fibroblasts in in vitro experiments, thereby demonstrating positive biological properties [42].

#### 7.1.3. Glycosaminoglycan (GAG)

Computer molecular modeling has made significant contributions to drug design and discovery. It can predict the physical and chemical properties of drug molecules, determine compound–receptor interactions, and evaluate drug effects at the molecular level by simulating drug-receptor interaction processes (docking) [124,125]. Recently, by using the computer Molegro Virtual Docker (MVD) program, Sularsih et al. found that the scaffold material formed by combining AC and GAG had osteogenic activity. This scaffold can bind to TLR-2 through hydrogen bonding and strong steric hindrance interactions. The signal compound regulated by TLR-2 can recognize the endogenous DAMP molecules released after alveolar bone injury, thereby activating macrophages to release important growth factors that promote alveolar bone healing and promoting bone formation [126,127,128].

### 7.2. Combining AC with Collagen (Col)

Col is the most widely distributed protein in connective tissue, and it plays an important role as a natural binding segment in tissue healing. It provides the biological microenvironment required for cell adhesion, proliferation, and migration in the process of tissue construction or repair and is considered a potential biomaterial for tissue engineering [129,130]. Previous studies have found that aloes have good biocompatibility with Col. Incorporating aloes into Col membranes increases its surface hydrophilicity, which leads to better fibroblast adhesion during wound healing and promotes tissue regeneration [131]. Thant found that adding AC to Col scaffolds to form AC/Col composite scaffolds enhanced pulp tissue regeneration. Compared with individual Col scaffolds, on the one hand, AC/Col scaffolds exhibited higher hydrophilicity, swelling, porosity, and larger pore diameter in terms of physical properties. On the other hand, AC/Col exhibited better biological activity. For example, through the use of electron microscopy, it was found that AC/Col scaffolds induced cell proliferation, enhanced adhesion between cells and cell scaffolds, and increased the expression of dentin extracellular matrix proteins [132].

### 7.3. Combining AC with Lipids

Research has found that AC can improve the drug loading effect of carriers. Stearic acid (SA) is a commonly used lipid matrix for drug delivery [133,134]. However, one of its main drawbacks is its highly crystalline nature, which makes it difficult to use as a matrix for manufacturing solid lipid nanoparticles (SLN). Therefore, in order to increase the drug loading efficiency of SA, Joshy et al. developed modified stearic acid nanoparticles containing AC, which synergistically acted with zidovudine to exert an antiviral effect. The results showed that the addition of AC can reduce the crystallinity of SLN and improve its hydrophilicity, enhancing the absorption of antiviral drugs in the brain [135]. In addition, Gomes et al. developed a bead containing AC, caffeate, and ALG, which greatly promoted drug encapsulation and the sustainable release of caffeate. Moreover, this bead, which is considered a promising solution for cartilage lesions, provided a suitable environment for the growth of ATDC5 chondrocyte-like cells and the formation of the chondroid extracellular matrix and blocked the secretion of pro-inflammatory cytokines by differentiated THP-1 [136].

### 7.4. Combining AC with Plants

For centuries, turmeric has had both edible and medicinal value. Curcumin extracted from the rhizome of turmeric has been used as an anti-inflammatory agent. In various experimental conditions and clinical environments, curcumin has been found to have antibacterial, antioxidant, antifungal, antiviral, and anticancer activities [137]. Sharma et al. found that the composite hydrogel formed by the combination of AC and curcumin can produce a synergistic effect in the treatment of skin wounds. On the seventh day, the wound was quickly closed, and on the tenth day, the hair was completely grown. However, when used alone, they both need more than 20 days of wound closure time [138]. However, Pachimalla et al. found that this composite hydrogel inhibited the biological activity of osteoblasts by wrapping it on titanium discs through in vitro experiments. It was also found that changing the hydrogel concentration could not create favorable conditions for cell proliferation. Moreover, an independent analysis of these two components found that AC had significantly better results, while curcumin had a very high cell inhibition rate [139]. Therefore, we do not recommend that this composite hydrogel be used for bone tissue regeneration, and the same conclusion has been drawn in previous studies. This may be an apoptosis-dependent mechanism of curcumin that inhibits the metabolism of osteoblasts and osteoclasts [140,141,142]. In addition, a new type of nanofiber scaffolds modified with AC can also be used for the treatment of tumors. For example, Ekambaram et al. found that titanium dioxide nanorods synthesized through AC mediation combined with PVA nanofibers loaded with resveratrol drugs can effectively eliminate free radicals and have a significant inhibitory effect on A431 skin cancer cells, as well as skin pathogenic bacteria Staphylococcus aureus and Pseudomonas aeruginosa, thus proving to be an effective skin cancer treatment material [143]. Pachimalla et al. implanted titanium discs coated with AC and Moringa hydrophilic gel into the tibia and femur bones of rabbits. The results showed that new bone formation occurred on the surface of the hydrophilic implant; bone–implant contact increased; and no degenerative changes, necrotic changes, fibrosis, or inflammation were observed. In addition, it was found in in vitro experiments that AC and Moringa water extract (2:1) had the weakest inhibitory effect on UMR106 cells [144]. Yahya et al. found that the preparation of mangosteen and aloe peel as nutritional health foods helps to control the blood sugar level of patients with diabetes. Through an experimental test of the content of compounds, it was found that the anti-hyperglycemic activity of this product was related to the flavones in the mangosteen and the AC and phytosterol in aloe [145].

### 7.5. Combining AC with Other Compounds

Yates et al. found that an antibacterial wound gel composed of a low concentration of proprietary silver salt and AC had inhibitory effects on six common wound pathogens, including Pseudomonas aeruginosa, methicillin-resistant Staphylococcus aureus, and Escherichia coli. Moreover, its effectiveness was comparable to or even better than that of other localized silver products with much higher silver concentrations [146]. Zhang et al. found that a hydrogel prepared with AC, honey, and PVA had good mechanical strength and biocompatibility and an obvious inhibitory effect on Staphylococcus aureus, Escherichia coli, and Candida albicans, which can accelerate the healing of infected wounds [39]. Furthermore, the combination of AC and other antiviral drugs can produce synergistic effects and enhance drug efficacy. For example, using AC in combination with zidovudine (AZT) or acyclovir (ACY) for antiviral treatment can inhibit the replication of HIV-1 and herpes simplex virus type 1. Moreover, the inhibitory effect of AC on the virus does not overlap with that of AZT or ACY and seems to be related to the glycosylation modification of viral glycoproteins [147]. The combined application of AC with other compounds is shown in Table 3.

## 8. Conclusions and Prospects

AC is the main polysaccharide component extracted from *Aloe vera*, and it plays an important role in transmitting information between cells and regulating bodily functions. In particular, in the past five years, many scholars have begun to attempt to incorporate AC into clinical research, which is nothing more than a breakthrough progress, laying the foundation for the future preparation of AC finished drugs and large-scale clinical use. However, in order to provide a scientific basis for the safer and more comprehensive use of AC in the treatment of diseases, there are still many aspects worthy of in-depth research and exploration. 

### 8.1. Using Computer-Aided Drug Design (CADD) Systems for Drug Efficacy Analysis

In clinical treatment, a large number of studies have compared the therapeutic effects of AC with positive drugs, but the conclusions drawn have been inconsistent. For example, in 2020, Vu demonstrated that the success rate of AC in pulpotomy was lower than that of the MTA group. However, Poor believed that AC had a better effect in promoting alveolar bone repair than gelatin sponge treatment. This prompts the question of what factors are related to the difference in efficacy between AC and positive drugs. In order to study the mechanisms by which different drugs exert their efficacy, CADD can be used. CADD relies on research achievements in life sciences such as biochemistry, enzymology, molecular biology, and genetics to design targets for potential drugs revealed in basic research, including enzymes, receptors, and nucleic acids. It also refers to the chemical structural characteristics of other class-derived ligands or natural products and uses computer chemistry as the foundation to simulate, calculate, and budget the interactions between drugs and receptor macromolecules through computer simulation. Among them, molecular docking is an important means in CADD. We assume that we can study the interaction between AC and positive drugs and protein macromolecules and predict their binding patterns through molecular docking. It is necessary to explore the difference of binding patterns between drugs and positive controls to determine whether drugs have the potential to become candidate drugs. 

### 8.2. Druggability Analysis of AC

Before any drug is put into large-scale clinical use, it is necessary to conduct druggability analysis to preliminarily determine whether the compound has the potential to be developed into a drug. Druggability refers to all of other properties of a given drug except for its activity, including the physical and chemical properties, biochemical properties, pharmacokinetics properties, and toxic side effects. The key to determining druggability is the multiple parameters of AC in vivo, including absorption, distribution, metabolism, excretion, and toxicokinetics. However, in the existing literature, few studies have reported the side effects of AC on tissue regeneration function. In future, we should emphasize the importance of conducting druggability analysis on AC and the publishing of pioneering research progress, as this will help to provide a theoretical basis for the production of AC in finished drugs for clinical use.

### 8.3. Toxicity Analysis of AC

It is well known that a new drug needs to undergo repeated toxicity and toxicological tests to verify its safety before it is put into clinical trials. At present, the toxicity analysis reports on AC are mostly positive, and no obvious adverse reactions or toxic effects have been found. For example, in a previous in vivo experiment evaluating the toxicology of AC, Fogleman et al. injected 1.0 mg/mL AC into mice, rats, and dogs at a single dose or eight repeated doses at 4 day intervals via the iv or ip routes. None of the results showed significant signs of toxicity or mortality. Additionally, during repeated injections, systemic toxicity was only limited to obvious temporary discomfort, which seemed to be dose-related [148]. In addition, on the 14th day, the no observed effect level (NOEL) for AC in the diet of rats was 50,000 ppm or 4.1 to 4.6 g/kg day (−1) [149]. In in vitro experiments, Jettanachewchankit et al. found that, even at a maximum concentration of 16 mg/mL, AC was not toxic to gingival fibroblasts, and AC significantly stimulated the proliferation of gingival fibroblasts and the expression of vascular endothelial growth factor (VEGF) [91]. However, it is worth noting that when AC is exposed to higher temperatures, a toxic reaction occurs, resulting in higher cytotoxicity. When AC is heat-treated at a temperature of 80 °C or higher, it triggers structural changes in AC, causing to its deacetylation and promoting cytotoxicity in human intestinal cells HT-29 [150]. Currently, studies on the toxicity of AC are scarce, and studies on the content and/or concentration of potential toxins and the genotoxicity, carcinogenicity, and adverse clinical effects of AC are also scarce; therefore, further systematic toxicity analyses are necessary to more fully explore the safety and efficacy of the bioactive polysaccharide.

### 8.4. Exploring the Relationship between Surface Structure and Molecular Structure and Drug Activity

Different treatment methods and degrees of deacetylation can lead to the formation of different surface structures of AC, mainly including granular, porous, and sheet-like structures. It is worth mentioning that even the same structure has slight differences. For example, AC aerogel made via different methods seemingly has a porous surface structure under an electron microscope. Among them, in aerogel made of acetone, we can see the obvious open porous structure at the lowest magnification, but a larger magnification is required for aerogel made by adding alkali to see the obvious porous structure, which indicates that the micropore diameter of the former is much larger than that of the latter. In addition, the surface structure of AC is also affected by deacetylation, changing from an original sponge-like structure with multiple three-dimensional connected pores to an irregular flat layered structure. However, there is no experiment to determine whether the functional changes pertaining to AC after deacetylation are related to changes in surface structure. In future research, more efforts should be made to explore the relationship between the surface structure, function, and activity of AC in order to determine the most suitable surface structure for AC to perform different functions.

### 8.5. Limitations of AC Application in Tissue Engineering

Clinical case reports on the application of AC in the tissue regeneration engineering of oral and maxillofacial tissues, including teeth, skin, mucosa, and alveolar bone, have opened up a new path but still have many shortcomings. On the one hand, the effectiveness of tissue repair is not outstanding. Tissue regeneration engineering includes three elements: seed cells, growth factors, and scaffold materials. Among them, growth factors play a key role in the repair process, accelerating the repair process and improving the repair effect. At present, there is very little research on the combined application of growth factors and AC. Scholars can innovatively add growth factors such as BMP and TGF to experiments designed to test AC’s efficacy in repairing tissue defects in future research to explore the combined effect of the two. On the other hand, the types of repair tissues have limitations. The oral and maxillofacial regions have a crucial joint structure called the temporomandibular joint, and temporomandibular joint disorders have been a long-standing problem for patients. One of the serious consequences of disease development is temporomandibular joint cartilage damage. There is currently no comprehensive therapeutic drug that can repair articular cartilage defects. Given that AC has been shown to have good effects in repairing alveolar bone, the authors speculate whether the addition of growth factors for cartilage repair can assist in the repair of articular cartilage defects. This will be a significant breakthrough and valuable research topic.

### 8.6. Developing and Perfecting the Application Form of AC

It is well known that the form of application regarding drugs directly affects their therapeutic effect; thus, further exploration is needed for the application form of AC. At present, the most commonly used forms are spongy, granular, and gel AC, each of which has its own unique characteristics. Sponge and granular drugs have a filling effect but poor resistance, making them unsuitable for repairing tissue defects in load-bearing areas. The gel drug has strong mobility but low efficacy maintenance time, so it must be administered many times. It can be seen that a comprehensive form of medication has not yet been developed for the repair of skin, mucosal, and large bone defects. Importantly, the study of AC materials can focus on the production of finished products in the form of patches or spray for the repair of small mucosal ulcers and skin defects, the production of finished dressings for repairing large-scale skin damage, and other aspects. 

In conclusion, polysaccharides in plants have many important functions in the human body, and AC derived from aloe has great potential with respect to scientific research. We expect that, through the joint efforts of scholars from various countries, the use of AC as a new biological material will facilitate breakthroughs in science, biology, chemistry, medicine, and pharmacology in the next few years.

## Figures and Tables

**Table 1:** Biological Function of acemannan (AC).

Function	Source	Dose/Form	Cell/Animal	Results	Ref
Immunomodulation	Freeze-dried gel	0.5% (solution)	T cells from human PBMC	Cytotoxic T cell generation ↑ Response to alloantigen ↑	[60]
	Freeze-dried gel	100 μg/mL (solution)	Immature dendritic cells (mouse)	Induced maturation of immature DCs MHC-II, B7-1, B7-2, CD40, CD54 ↑	[61]
	Fresh gel	1–8 mg/mL (hydrogel)	Macrophage cells (rats)	Macrophage cell activity ↓ Accelerated wound healing	[62]
	Freeze-dried gel	0.5–5.0 mg/mL (gel)	T cells from human PBMC	T cell activation and proliferation ↓ IL-2, IL-5, IL-17 ↓	[63]
	Fresh gel	2 mg/mL (solution)	Splenocytes and macrophages (chicken)	NO ↑	[65]
	Fresh gel	100 μg/mL (solution)	RAW 264.7 cells (mouse)	Activation of macrophages ↑ IL-6, TNF-ɑ, NO ↑	[66]
	Fresh gel	500 μg/mL (solution)	Macrophage cells (chicken)	Activation capacity of macrophages ↑ NO ↑	[67]
	Fresh gel	1–2 mg/mL (solution)	Hematopoietic progenitors (mouse)	Hematopoietic activity ↑	[68,69]
	Fresh gel	50 mg/kg (pellet)	Splenocytes (mouse)	Peripheral lymphocyte counts; spleen cellularity; spleen index ↑ TNF-ɑ; IL-1 ↑	[70]
Antiviral effect	Fresh gel	31.25–62.5 mg/mL (solution)	CEM-SS1 and MT-2(2) cells	Inhibited viral replication	[72]
	Fresh gel	2–100 mg/kg (solution)	Lymphocytes (cats)	Lymphocyte counts ↑	[73]
	Lyophilized powder	0.5 mg/kg (solution)	Mouse	Anti-CVB3m antibody titers ↑	[76]
	Lyophilized powder	0.1–0.5 mg/mL (solution)	Chicken	Protective antibody titers ↑	[77]
Anti-tumor effect	Fresh gel	Solution	Macrophages (mouse)	IL-1; TNF ↑	[79]
	Fresh gel	200–400 mg/kg (gel)	Mouse	Activation of nuclear factor kappa B (NF-κB) ↓	[80]
	Fresh gel	-	Mouse	Regulated Bax and cytochrome-c mediated mitochondrial pathway.	[81]
Regeneration of dental tissue	Fresh gel	0.25–1 mg/mL (solution)	Periodontal fibroblasts and pulpal fibroblasts	BMP-2 ↑	[84]
	Fresh gel	1–8 mg/mL (sponge)	Dental pulp cells (rats)	Cell; BMP-2 ↑ Produced homogeneous calcified dentin bridge and pulp tissue.	[85]
	Fresh gel	0.5–4 mg/mL (Sponge)	Dental pulpal cells (rats)	Cell; ALP; COL-1; BMP-2; BMP-4; VEGF; DSP ↑ Produced mineralized bridge with normal pulp tissue.	[86]
	Fresh gel	0.5–8 mg/mL (solution)	Cementoblasts	Cell; COL-1; VEGF; OPN; ALP ↑	[87]
Bone formation	Fresh gel	2–8 mg/mL (sponge)	BMSCs (rats)	Cell; VEGF; BMP-2; ALP; BSP; OPN; mineralization ↑ Induced bone formation.	[88]
	Fresh gel	Sponge	Rats	Increased bone surface, bone volume and tissue mineral density.	[35]
	Fresh gel	0.25–4 mg/mL (sponge)	PDLCs (dogs)	Cell; RUNX2; GDF5; VEGF; BMP2; COL-1; ALP; mineral deposition ↑ Accelerated alveolar bone, cementum and periodontal ligament formation.	[38]
Soft tissue healing	Fresh gel	150 μg/mL (solution)	Skin fibroblasts (mouse)	Cell ↑ Activation of AKT/mTOR.	[89]
	Fresh gel	0.01–10 mg/mL (solution)	Human gingival fibroblasts	Induced NF-ĸB/DNA binding and IL-6/-8 expression via Toll-like receptor-5.	[90]
	Fresh gel	2–16 mg/mL (solution)	GFs (rats)	Cell; KGF-1; VEGF; COL-1 ↑	[91]
	Fresh gel	25–75% (hydrogel)	Rats	Increased collagen density in gingival tissue.	[40]
	Fresh gel	Gel	Sheep	Dehydrated the wounds and stimulated late granulation tissue and cell proliferation.	[92]

↑: increased; ↓: decreased.

**Table 2:** Advances in the clinical application of AC.

Clinical Application	Year	Application Field	Sample Size (Unit)	Follow-Up Time	Form/Dose	Control Group	Results	Ref
Regeneration of pulp–dentin complex	2022	Apexogenesis	2 (people)	12 months	Sponge	-	Preserved pulp vitality and form apical stop.	[100]
	2020	Pulpotomy (young permanent teeth)	50 (tooth)	12 months	Sponge	MTA	Induced continued root formation.	[99]
	2017	Pulpotomy (deciduous teeth)	46 (tooth)	12 weeks	Sponge	FC	Promoted dentin bridge formation.	[98]
	2015	DPC (deciduous teeth)	42 (tooth)	6 months	Sponge (0.4 mg)	calcium hydroxide	Promoted dentin bridge and soft tissue formation.	[36]
Bone formation	2023	Periodontal surgery	3 (tooth)	5 years	Sponge	-	Reduced probing pocket depth, increased clinical attachment level, and bone density.	[110]
	2022	GBR	20 (people)	6 months	Particle	DBB	Enhanced dimensional stability of the regenerated tissue.	[107]
	2021	Alveolar ridge preservation	35 (people)	12 months	Sponge (20, 50 mg)	Spontaneous blood-clotting	Reduced tooth socket volume.	[101]
	2020	Lateral sinus augmentation	1 (people)	6 months	Sponge (150 mg)	-	Increased bone height significantly.	[105]
	2019	Indirect sinus augmentation	30 (people)	6 months	Sponge (50 mg)	No-treatment control	Enhanced endo-sinus bone formation greatly.	[34]
	2019	Apical surgery	22 (tooth)	12 months	Sponge (5, 10 mg)	Spontaneous blood-clotting	Enhanced early bone healing.	[102]
	2018	Periodontitis with furcation defects	90 (people)	12 months	Gel	ALN	Improved periodontal pocket depth and attachment loss.	[108]
	2016	Alveolar ridge preservation	99 (people)	3 months	Sponge (50 mg)	Spontaneous blood-clotting	Increased the bone density and tooth socket healing.	[37]
	2002	Alveolar osteitis	1194 (people)	7 days	Hydrogel	Gelfoam	Reduced the incidence of alveolar osteitis.	[103]
Treatment of skin and mucosal diseases	2013	Aphthous ulcer	100 (people)	7 days	Gel (0.5%)	Triamcinolone	Reduced ulcer size and pain.	[111]
	1998	Pressure ulcers	30 (people)	10 weeks	Hydrogel	Moist saline gauze	Promoted ulcer healing.	[114]
Improvement of blood sugar and lipids	2011	Advanced Type 2 Diabetes	35 (people)	2 months	Capsule (300 mg)	Placebo capsules	Lowered the blood levels of fasting glucose and glycosylated hemoglobin significantly.	[116]
	2011	Hyperlipidemic Type 2 Diabetes	67 (people)	2 months	Capsule (300 mg)	Placebo capsules	Lowered the fasting blood glucose, HbA1c, total cholesterol, and LDL levels significantly.	[117]

**Table 3:** Combined application of AC with other compounds.

Drug	Application Field	Application Form	Result/Function	Ref
AC + CS	Wound healing	Hydrogel	Weakened the mechanical strength and biological activity of the CS gel with increasing AC.	[118]
AC + CS	Wound healing	Film	Resulted in strong synergistic effects and leaded to mixed junction zones formation.	[41]
AC + CS	Osseointegration	Solution	Improved osseointegration with a seamless implant interface.	[119]
AC + CS	Carrier modification	Solution	Enhanced drug loading capacity.	[120]
AC + CS	Tissue regeneration	Scaffold	Repaired tissue defects caused by ameloblastoma.	[121]
AC + ALG	Wound healing	Film	Leaded to more resistant and stable structures.	[41]
AC + ALG	Antioxidation	Film	Enhanced the antioxidant capacity.	[123]
AC + ALG + CS	Wound healing	Film	Retained and created a moist environment around the wound to promote its healing.	[42]
AC + GAG	Bone healing	Scaffold	Binded with a TLR-2 target receptor.	[126]
Ac + COL	Pulp regeneration	Scaffold	Increased expression of dentin extracellular matrix proteins.	[132]
AC + SA	Carrier modification	Nanoparticle	Improved hydrophilicity to enhance drug absorption.	[135]
AC + Caffeate + ALG	Treatment of osteoarthritis	Bead	Promoted ATDC5 chondrocyte-like cell growth and cartilage-like extracellular matrix formation.	[136]
AC + Curcumin	Wound healing	Hydrogel	Reduced wound healing days greatly.	[138]
AC + Curcumin	Osseointegration	Hydrogel	Inhibited osteoblast differentiation.	[139]
AC + Resveratrol	Treatment of skin cancer	Scaffold	Inhibited the growth of A431 skin cancer cells and skin pathogens (e.g., *Staphylococcus aureus* and *Pseudomonas aeruginosa*).	[143]
AC + Moringa oleifera	Osseointegration	Hydrophilic gel	Increased bone contact with implant.	[144]
AC + Silver salt	Wound healing	Gel	Showed broad-spectrum antimicrobial activity.	[146]
AC + Honey	Wound healing	Hydrogel	Inhibited the growth of *Staphylococcus aureus, Escherichia coli*, and *candida albicans*.	[39]
AC + AZT or ACY	Antiviral	-	Inhibited the replication of HIV-1 and HSV-1.	[147]

## Data Availability

Not applicable.

## Abbreviations

AC	Acemannan
CS	Chitosan
DeAC	Deacetylated acemannan
FD	Freeze-drying
IFD	Industrial freeze-drying
SD	Spray-drying
RWD	Refractance window drying
RZD	Radiant zone drying
SEM	Scanning electron microscopy
PDLCs	Periodontal ligament cells
PVA	Polyving akohol
LPS	Lipopolysaccharide
TCs	T cells
NO	Nitric oxide
INF-γ	Interferon-γ
TNF-α	Tumor necrosis factor-α
IL-1	Interleukin-1
HIV	Human immunodeficiency virus
FIV	Feline immunodeficiency virus
BRM	Biological response modulators
CVB3	Coxsackievirus B3
NDV	Newcastle disease virus
IBDV	Infectious bursal disease virus
PAG	processed *Aloe vera* gel
BMP-2	Bone morphogentic protein-2
MTA	Mineral trioxide aggregate
PDPCs	Primary human dental pulp cells
TLR-2	Toll-like receptor 2
COL-1	Type I collagen
FC	Formocresol
BMSCs	Bone marrow mesenchymal stem cells
Runx2	Runt-related transcription factor 2
GDF-5	Growth differentiation factor 5
DPC	Direct pulp capping
CBCT	Cone-beam computed tomography
GBR	Guided bone regeneration
DBB	Deproteinized bovine bone
ISQ	Implant stability quotient
AV	*Aloe vera*
ALN	Alendronate
mSBI	Modified gingival sulcus bleeding index
GTR	Guided tissue regeneration
GAG	Glycosaminoglycan
ALG	Alginate
Col	Collagen
SA	Stearic acid
SLN	Solid lipid nanoparticles
AZT	Zidovudine
ACY	Acyclovir
CADD	Computer aided drug design
NOEL	No observed effect level
